# MRI evidence for material‐specific encoding deficits and mesial–temporal alterations in presurgical frontal lobe epilepsy patients

**DOI:** 10.1002/epi4.12881

**Published:** 2024-01-04

**Authors:** Anna Doll, Martin Wegrzyn, Friedrich G. Woermann, Kirsten Labudda, Christian G. Bien, Johanna Kissler

**Affiliations:** ^1^ Department of Epileptology (Krankenhaus Mara) Medical School, Bielefeld University Bielefeld Germany; ^2^ Department of Psychology Bielefeld University Bielefeld Germany; ^3^ Center for Cognitive Interaction Technology (CITEC) Bielefeld University Bielefeld Germany

**Keywords:** fMRI, frontal lobe epilepsy, memory encoding, structural changes

## Abstract

**Objective:**

Neuroimaging studies reveal frontal lobe (FL) contributions to memory encoding. Accordingly, memory impairments are documented in frontal lobe epilepsy (FLE). Still, little is known about the structural or functional correlates of such impairments. Particularly, material specificity of functional changes in cerebral activity during memory encoding in FLE is unclear.

**Methods:**

We compared 24 FLE patients (15 right‐sided) undergoing presurgical evaluation with 30 healthy controls on a memory fMRI‐paradigm of learning scenes, faces, and words followed by an out‐of‐scanner recognition task as well as regarding their mesial temporal lobe (mTL) volumes. We also addressed effects of FLE lateralization and performance level (normal vs. low).

**Results:**

FLE patients had poorer memory performance and larger left hippocampal volumes than controls. Volume increase seemed, however, irrelevant or even dysfunctional for memory performance. Further, functional changes in FLE patients were right‐sided for scenes and faces and bilateral for words. In detail, during face encoding, FLE patients had, regardless of their performance level, decreased mTL activation, while during scene and word encoding only low performing FLE patients had decreased mTL along with decreased FL activation. Intact verbal memory performance was associated with higher right frontal activation in FLE patients but not in controls.

**Significance:**

Pharmacoresistant FLE has a distinct functional and structural impact on the mTL. Effects vary with the encoded material and patients' performance levels. Thus, in addition to the direct effect of the FL, memory impairment in FLE is presumably to a large part due to functional mTL changes triggered by disrupted FL networks.

**Plain Language Summary:**

Frontal lobe epilepsy (FLE) patients may suffer from memory impairment. Therefore, we asked patients to perform a memory task while their brain was scanned by MRI in order to investigate possible changes in brain activation during learning. FLE patients showed changes in brain activation during learning and also structural changes in the mesial temporal lobe, which is a brain region especially relevant for learning but not the origin of the seizures in FLE. We conclude that FLE leads to widespread changes that contribute to FLE patients’ memory impairment.

**Key points**

- Presurgical frontal lobe epilepsy (FLE) patients have verbal and nonverbal memory impairments.
- Presurgical FLE patients have increased left hippocampal volumes.
- Presurgical FLE patients have decreased hemodynamic activation during encoding especially in the mesial–temporal lobe.

## 1 INTRODUCTION

In temporal lobe epilepsy (TLE), memory impairments have been extensively investigated, as the temporal lobe (TL) is closely associated with memory formation. However, studies suggest that patients with frontal lobe epilepsy (FLE), the second most common type of focal pharmacoresistant epilepsy,^1^ are also prone to memory deficits.^2^ In fact, a recent behavioral study comparing large and well‐matched groups of TLE and FLE patients found no difference in the extent of verbal memory impairment between groups, neither pre nor postoperatively.^3^ In line with memory impairment in FLE, current theory suggests that the memory network comprises frontal and parietal regions in addition to TL structures.^4^ The importance of frontal lobe (FL) structures for memory is underlined by neuroimaging studies^5^ which reveal their involvement in both encoding and retrieval. Overall, studies suggest a material‐specific impact with word encoding being associated with primarily left frontal activation, while face encoding is associated with more right than left frontal activation.^6^ Scene encoding typically elicits bilateral to slightly right‐lateralized frontal activation.^7^

First neuroimaging studies on FLE hint not only at structural and functional disruptions in the FL itself. They further indicate that FLE patients might have structural and functional disruptions in core memory structures distal to the FL, especially the mesial TL (mTL).^8^, ^9^

Structural changes in FLE patients were found in terms of volume reductions of white matter bundles^10^ and cortical thinning^11^ in various regions. Regarding TL structures, studies showed reduced left lateral TL^12^ and increased bilateral mTL volumes.^9^ In contrast, other studies did not find mTL volume changes.^8^, ^13^ To the best of our knowledge, none of these studies correlated structural changes with memory performance.

In addition to investigating structural changes, it is informative to study functional changes in FLE patients to elucidate the contribution of the FL to memory. This may also help to predict postoperative memory impairment in a similar way as in TLE.^14^, ^15^, ^16^ To the best of our knowledge, there is only one study investigating functional magnetic resonance imaging (fMRI) correlates of episodic memory in FLE patients.^8^ This study compared 18 controls with 32 predominantly nonlesional FLE patients (19 left, 13 right, eight with established frontal lesions) regarding encoding of line‐drawn objects, faces and words. Across stimulus types, FLE patients showed more frontal activation than controls, especially contralateral to seizure focus. Further analyses revealed that these frontal activation increases were restricted to patients with normal memory performance. By contrast, patients with low memory performance showed decreased hippocampal activation.

Here, we study possible functional and structural impacts of FLE on core memory structures, including the mTL. Going beyond a previous study we investigate whether and how fMRI activation in FLE patients varies with the type of encoding material. Further, we examine the association between memory performance and both structural changes and functional activation in the mTL. Therefore, we applied a memory fMRI‐paradigm of learning scenes, faces and words, that we previously used to elicit robust activations throughout the brain including the mTL,^17^ in a group of FLE patients with predominantly structural etiology undergoing presurgical evaluation.

## 2 METHODS

### 2.1 Participants

We studied 25 FLE patients, who were consecutively recruited from the presurgical epilepsy monitoring unit at The Mara, Bielefeld, Germany, and 30 controls (largely overlapping with the previously published control sample^17^). One patient who reported to have fallen asleep during the experiment was excluded. Two additional patients were excluded from the verbal encoding condition only: one right FLE (rFLE) patient, because of deficient German, and one left FLE (lFLE) patient who reported not having seen the words but only scenes and faces clearly.

FL origin and lateralization of the patient's epileptic onset zone were ascertained by presurgical diagnostics (using semiology, MRI, noninvasive long‐term‐video‐EEG‐monitoring [about 1 week], and if available invasive recordings). Requirements for controls’ participation were an age ≥18 and absence of known neurological and psychiatric disorders. Table 1 details participants’ demographic and clinical characteristics.

**TABLE 1:** Demographic and clinical characteristics of controls and FLE patients.

	Controls *n* = 30	FLE *n* = 24
Age in years [*M* (SD) (range)]	35.4 (13.3) (19–60)	32.5 (12.7) (18–65)
Sex in % [male/female]	50.0/50.0	58.3/41.7
Years of schooling [*M* (SD)] (range)	10.6 (1.6) (9–13)	10.8 (1.4) (9–13)
Handedness [right/left]	28/2	22/2
Language laterality^a^ [left/bilateral]		22/2
Laterality of epileptic focus [left/right/bilateral]		8/15/1
Age at epilepsy onset [*M* (SD)] [(range)]		15.1 (14.9) (0–62)
Epilepsy duration [*M* (SD)] [(range)]		17.4 (9.2) (0–34)
Antiseizure medication load^b^ [Mdn (range)]		2.6 (1.0–5.4)
Etiology [absolute numbers (lFLE)] Focal cortical dysplasia/unspecified dysplastic lesion/cyst/cavernoma/tumor/no lesion		13 (4)/ 4 (1)/ 1 (0)/ 2 (1)/ 2 (1)/ 2 (1)
Surgical outcome^c^ no surgery/1a/3a		8/14/2

Note: There was no statistically significant difference between controls and FLE patients regarding age, sex, years of schooling and handedness (ps > .3). The effect of language laterality and etiology on the outcome measures was explored in Supplementary Information V in the Appendix S1.

Abbreviation: FLE, frontal lobe epilepsy.

^a^ Language laterality was determined by language fMRI.^18^

^b^ The antiseizure medication load was estimated as the sum of the ratios (prescribed daily dose/defined daily dose) for each antiseizure medication, with the doses corresponding to those defined by the World Health Organization. One patient took 100 mg/day Topiramate, none took Zonisamide.

^c^ According to Engel^19^ at 6‐month (9 patients) or 24‐month after surgery (7 patients).

Before participation, all participants gave written informed consent according to the Declaration of Helsinki. The ethics committees of Bielefeld University (EUB 2017‐080) and Westphalia‐Lippe Medical Association (Münster, 2018‐090‐f‐S) approved the study.

### 2.2 Neuropsychological testing

Participants underwent neuropsychological testing including verbal and visual memory assessment using the German adaption of the Rey Auditory Verbal Learning and Memory Test^20^ (Verbaler Lern‐ und Merkfähigkeitstest,^21^ VLMT) and the Diagnosticum für Cerebralschädigung II^22^ (DCS), respectively. In the VLMT, participants listened to five repetitions of the same list of 15 unrelated words, which had to be freely recalled immediately after each presentation. Then, participants listened to an interference list, which had to be freely recalled, too. Immediately afterwards and again after a 30‐min delay participants had to freely recall the primary list again. In the DCS, participants were presented 9 geometrical figures 5 times for 10 s each and, after each presentation, asked to reproduce the memorized figures with wooden sticks. For both tests, the standardized, age‐(and education‐) corrected *z*‐scores of the sum of recalled items across five trials were calculated according to published norms. Only for the VLMT, the sum of recalled items after 30 min was also assessed.

### 2.3 Memory fMRI‐paradigm

The memory fMRI‐paradigm comprised 72 scenes, faces and words, respectively, for details, see ref. [17] and Table S1. In three randomized consecutive runs, the stimuli of each condition were presented for 3 s each in alternating blocks of four neutral or negative stimuli. Each block was followed by a 12 s baseline condition. Participants were instructed to memorize the stimuli for subsequent recognition. In the baseline condition, participants were requested to maintain fixation at a randomly moving dot and to avoid thinking about the preceding stimuli.

The out‐of‐scanner recognition task immediately followed the fMRI‐task. For each condition all 72 old and 48 new stimuli were presented and participants had to decide whether the stimulus had been shown in the MRI.

### 2.4 MRI acquisition and preprocessing

MRI data were collected on a 3T Siemens Verio MRI scanner. For fMRI, 37 coronal slices aligned with the long axis of the hippocampus were acquired. We preprocessed MRI data using fMRIPrep 1.4.1rc4.^23^ For further details on anatomical and functional MRI data acquisition and preprocessing ref.^17^

### 2.5 Statistical analyses

#### 2.5.1 Behavioral data

We compared FLE patients with controls regarding percentage of hits and false alarms, recognition accuracy (hits – false alarms) and response bias (false alarms/[100 – recognition accuracy])^24^ of the recognition task following fMRI separately for each condition and regarding verbal learning, delayed verbal recall and design learning. Because data were partly nonnormally distributed, we used the Mann–Whitney‐*U*‐test to compare groups (*p*_two‐sided_ ≤ .05). We did not correct for multiple comparisons in the behavioral data, due to the exploratory character of the study, because of the given sample size, and the general flexibility for choosing the number of tests to adjust for, which makes correction often arbitrary and thus spurious.^25^, ^26^

#### 2.5.2 mTL volumes

Hippocampal and amygdala volumes were estimated using recon‐all from Freesurfer 6.0.1 (http://surfer.nmr.mgh.harvard.edu/) as implemented in the fMRIprep pipeline.^27^, ^28^ We accounted for possible differences in whole brain volumes using the region‐to‐total intracranial volume ratio [respective brain volume in mm^3^/total intracranial volume *100]. To explore associations between mTL volumes and memory performance, we calculated correlations and further compared memory performance between patients with normal and abnormal hippocampal volumes according to the 84th percentile of controls.

#### 2.5.3 fMRI data

For details on the first level analysis, see ref. [17]. In short, these were conducted using the Python package Nistats 0.0.1b1. Regressors of interest were the three stimulus modalities, each convolved with the canonical double gamma hemodynamic response function.

For the second level analyses, one‐sample *t*‐tests were used to examine main effects of encoding elicited by each modality separately for FLE patients and controls and two sample *t*‐tests to compare groups within each modality. We also exploratively compared lFLE and rFLE patients with controls. FSL 6.0 (FMRIB Software Library) randomize 2.9 nonparametric permutation testing was applied with 10 000 permutations.^29^ All results were corrected for multiple comparisons using threshold‐free cluster enhancement (TFCE)^30^ and *p* ≤ .05 family‐wise error (FWE) rate, which is a conservative correction compared to other methods (e.g., false discovery rate or arbitrary uncorrected thresholds). Due to the exploratory character of the study, we additionally report *p*_(FWE)_ ≤ .1 values (denoted as tendencies) to give a more comprehensive picture of the activation pattern and ensure a certain level of sensitivity while still being aware of the expected FWE‐rate.^31^, ^32^ TFCE‐corrected values of *p*_(FWE)_ ≤ .1 lie roughly between *p*_uncorrected_ < .001 and *p*_uncorrected_ < .00001. We conducted whole‐brain and small volume correction (SVC) analyses for a mTL region of interest (ROI) encompassing the hippocampus and the amygdala as defined in the Harvard‐Oxford‐Atlas because of the often low signal‐to‐noise ratio in this region.^33^, ^34^, ^35^ Furthermore, we analyzed anatomically defined mTL and functionally defined frontal ROIs, using the Mann–Whitney‐*U*‐test. Functionally defined frontal ROIs were used, as anatomical frontal ROIs did not cover functional activation well. We used meta‐analytic maps from Neurosynth (neurosynth.org^36^) to generate functional frontal ROIs using the keywords: “pictures”, “face” and “words”. These maps are based on data of many studies and thus represent typical activations from thousands of individuals. To obtain symmetrical frontal ROIs, we swapped the extracted right FL activations (thresholded at *p*_Bonferroni_ = .05) to the left hemisphere. This resulted in two frontal clusters for scenes, referred to as orbitofrontal and inferior frontal cluster, one cluster for faces and one for words, see Figure 3C.

**FIGURE 1:**
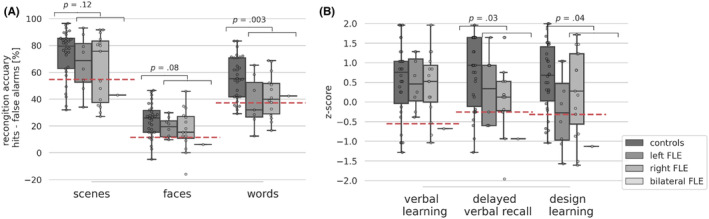
Recognition accuracy. Box plots with overlaid swarm plots, showing (A) the median recognition accuracy (hits – false alarms) for scenes, faces, and words in controls and FLE patients as well as within and between‐group variability and (B) verbal learning, delayed verbal recall and design learning *z*‐scores. Red dashed line indicates the 16th percentile of the controls' performance used for splitting patients into subgroups. *p*‐values (uncorrected) correspond to the comparison of controls and all FLE patients. Subgroups are shown for visual inspection only. Note that there was only one patient with bilateral FLE whose scores are indicated by a dot overlaid by a horizontal line. FLE, frontal lobe epilepsy.

Additionally, we explored potential functional relevance of brain regions. Firstly, we compared normal and low performing FLE patients and controls to facilitate comparison with a previous study.^8^ Therefore, the group of patients was split according to their recognition accuracy in each modality. We used the 16th percentile of memory performance in controls as reference (see Figure 1). Phenotyping using neuropsychological testing results was only applicable for the delayed verbal recall (for which we investigated differences in word and scene encoding activation), because only three patients performed below 16th percentile in verbal learning. Secondly, we examined correlations of brain activation and recognition accuracy and VLMT scores in patients and controls. Design learning scores did not correlate with scene, face or word recognition accuracy in either FLE patients or controls. Thus, we suppose design learning to be a distinct subfunction of nonvisual memory compared to scene and face recognition, which might be due to the differences in stimulus material, retrieval type (recall vs. recognition), or time of retrieval. Therefore, we concluded that the correlation of design learning scores with scene, face or word encoding activation would not be meaningful.

## 3 RESULTS

### 3.1 Behavioral data

FLE patients had significantly lower percentage of hits and recognition accuracy for words, a lower percentage of hits for scenes and in tendency a lower face recognition accuracy. The percentage of hits, false alarms, the recognition accuracy and the response bias are detailed in Table S2. The recognition accuracy is further depicted in Figure 1A. Further, FLE patients scored worse than controls in delayed verbal recall and design learning (Figure 1B, Table S3). An explorative comparison of lFLE and rFLE revealed no significant difference in memory performance. The percentage of impaired patients according to the 16th percentile criterion were: scene recognition 46%, face recognition 29%, word recognition 41%, verbal learning 12.5%, delayed verbal recall 25%, design learning 37.5%. *χ* ^2^ test indicated no impact of laterality.

### 3.2 mTL volumes

**FIGURE 2:**
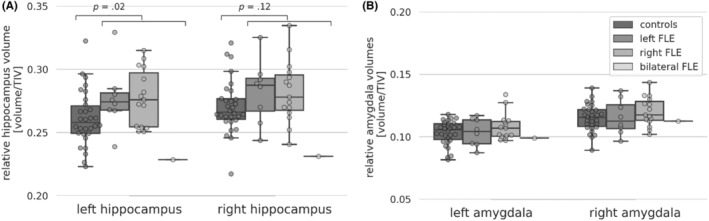
Mesial temporal lobe volumes. Box plot diagrams with overlaid swarm plots, illustrating the relative brain volume in mm^3^ in controls and FLE patients. Shown are median values as well as within and between‐group variability. (A) Relative hippocampal volumes. (B) Relative amygdala volumes. *p*‐values (uncorrected) correspond to the comparison of controls and FLE patients. Note that there was only one patient with bilateral FLE. FLE, frontal lobe epilepsy; TIV, total intracranial volume.

Left hippocampal volumes of FLE patients (Mdn = 0.27, range: 0.23–0.33) were significantly larger than controls' (Mdn = 0.26, range: 0.22–0.32, *U* = 231.0, *p* = .03, *d* = 0.64). No further significant differences between FLE patients and controls were observed, see Figure 2. We explored memory performance in FLE subgroups with normal and high hippocampal volumes. For the left hippocampus, no difference occurred. Patients with high right hippocampal volume had significantly lower word recognition accuracy (Mdn = 28.5, range: 12.5–58.3) and in tendency lower design learning scores (Mdn = −0.66, range: −1.61–1.04) than patients with normal right hippocampal volume (word recognition: Mdn = 47.2, range: 25.7–68.7, *U* = 22.0, *p* = .01, *d* = 1.28; design learning: Mdn = 0.61, range: −1.57–1.72, *U* = 41.5, *p* = .08, *d* = 0.77). In correlation analyses higher left (*r*_Spearman_ (22)= − .34, *p* = .10) and right hippocampal volumes (*r*_Spearman_ (22)= − .34, *p* = .11) tended to correlate with worse design learning scores in FLE patients. In controls, by contrast, higher bilateral hippocampal volumes were associated with better verbal learning (left hippocampus: *r*_Spearman_ (28) = .37, *p* = .04; right hippocampus *r*_Spearman_ (28) = .52, *p* = .003) and delayed verbal recall scores (left hippocampus: *r*_Spearman_ (28) = .32, *p* = .09; right hippocampus *r*_Spearman_ (28) = .43, *p* = .02) and higher right hippocampal volumes were in tendency associated with better word recognition (*r*_Spearman_ (28) = .30, *p* = .08). These correlation coefficients in controls differed significantly from FLE patients', whose correlations were consistently negative.

### 3.3 Memory fMRI activation

**FIGURE 3:**
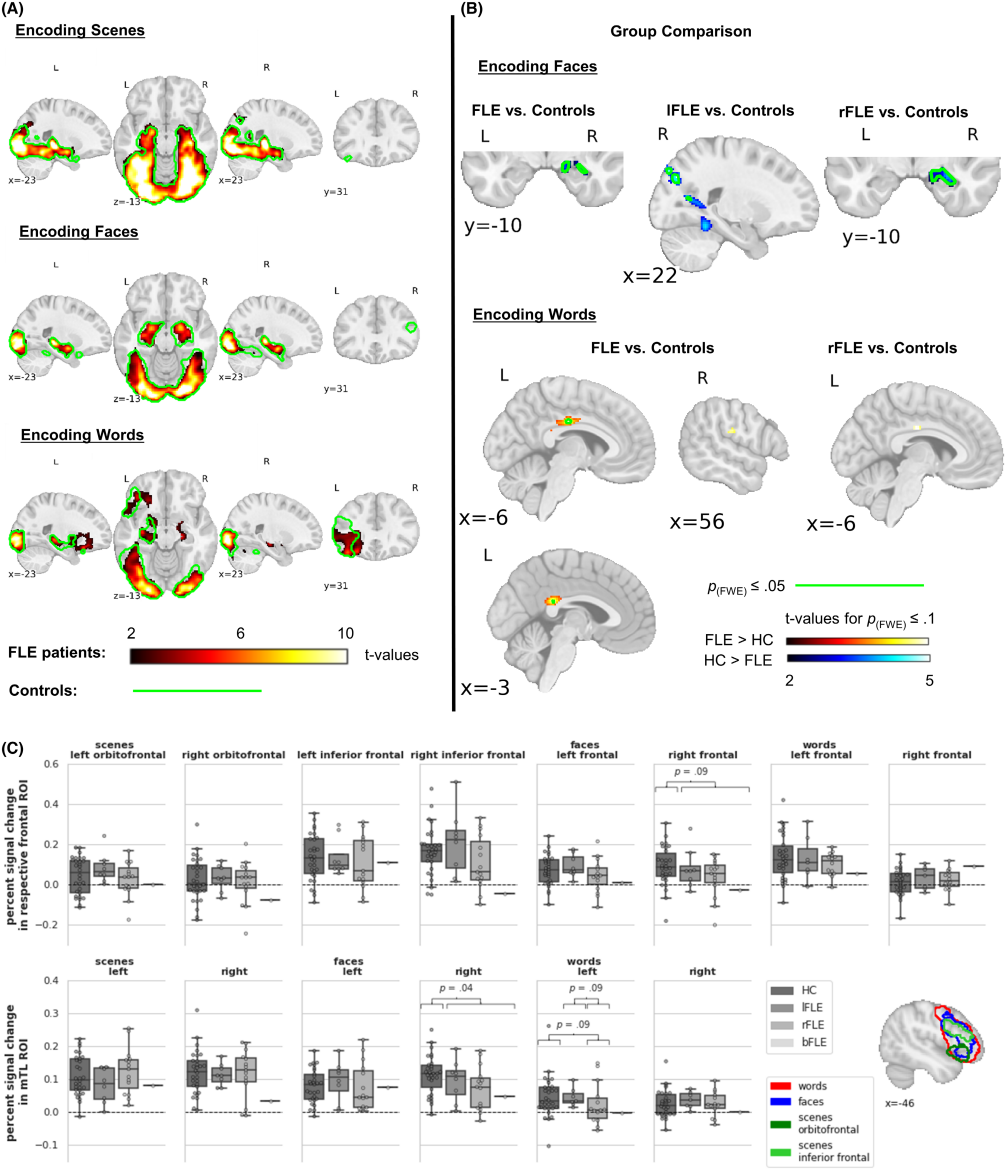
fMRI activation encoding versus baseline. (A) Overall fMRI activation of encoding scenes, faces, and words in FLE patients and controls. The color code indicates *t*‐values significant at *p*_(FWE)_ ≤ .05 of FLE patients. The green outline indicates *p*_(FWE)_ ≤ .05 of controls. (B) Group comparison of FLE patients and controls of the fMRI activation of encoding faces and words. We found no significant group difference for scene encoding. The color code indicates *t*‐values significant at *p*_(FWE)_ ≤ .1 and the green outline indicates *p*_(FWE)_ ≤ .05, both with SVC for the mesial temporal lobe. Data are displayed in MNI‐152 space. (C) ROI analyses. Box plot diagrams with overlaid swarm plots. Shown are median values as well as within and between‐group variability. *p*‐Values (uncorrected) correspond to Mann–Whitney‐*U*‐test. Note that there was only one patient with bilateral FLE. ROIs are illustrated via colored outlines on an MNI template in the right‐bottom box. ROI scenes: orbitofrontal cluster encompasses: orbitofrontal cortex, insula, frontal and central operculum, inferior frontal gyrus, temporal and frontal pole; and inferior frontal cluster encompasses: middle and inferior frontal gyrus, precentral gyrus and frontal pole; ROI faces encompasses: middle and inferior frontal gyrus, orbitofrontal cortex, precentral gyrus, insula, frontal operculum, frontal and temporal pole; ROI words encompasses: middle and inferior frontal gyrus, orbitofrontal cortex, temporal and frontal pole, precentral gyrus, frontal and central operculum, insula and putamen. bFLE, bilateral frontal lobe epilepsy; FLE, frontal lobe epilepsy; fMRI, functional magnetic resonance imaging; HC, healthy controls; lFLE, left frontal lobe epilepsy; and rFLE right frontal lobe epilepsy.

Figure 3A illustrates encoding activation of FLE patients and controls. Across stimulus types, FLE patients' encoding activation encompassed widespread bilateral temporal, basal ganglia, and occipital activations, which were largest during scene encoding. During word encoding, overall activation appeared more left‐lateralized with additional left frontal activation.

In controls, functional activation differed descriptively from patients especially regarding the FL. During scene and face encoding controls additionally exhibited clusters of frontal activation, and during word encoding frontal activation was more widespread (Figure 3).

### 3.4 Between‐group comparison

The statistical comparison of encoding activation between FLE patients and controls is detailed in Figure 3B,C and Table S4. Groups did not differ significantly during scene encoding. During face encoding, FLE patients exhibited significantly less activation than controls in the right anterior mTL. This was replicated only for the subsample of rFLE patients, suggesting that rFLE patients drove this effect. lFLE patients instead had reduced activation in bilateral occipital regions. Furthermore, FLE patients tended to have decreased activation in the right frontal ROI. During word encoding, only rFLE patients tended to have decreased activation in the left mTL ROI. Moreover, FLE patients demonstrated stronger activation than controls in the cingulate gyrus and in tendency in the right postcentral and opercular regions.

### 3.5 Normal‐ versus low‐performing FLE patients and controls

**FIGURE 4:**
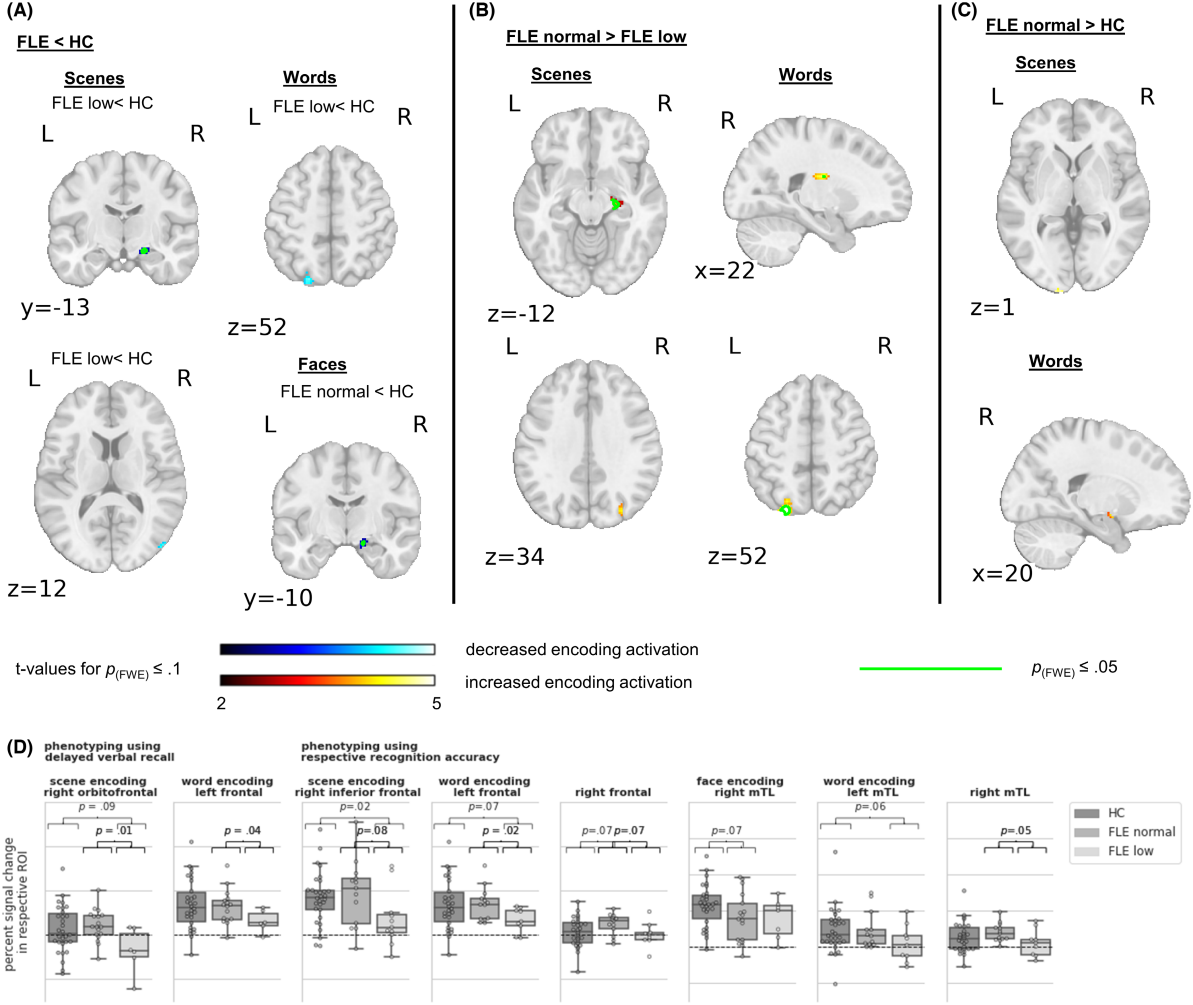
Group comparison of normal‐ and low‐performing FLE patients' and controls' encoding activation. (A) Normal and low performing FLE patients each with decreased activation compared to controls. (B) Normal compared to low performing FLE patients. (C) Normal performing FLE patients with increased activation compared to controls. The color code indicates t‐values significant at *p*_(FWE)_ ≤ .1 and the green outline indicates *p*_(FWE)_ ≤ .05, both with SVC for the mesial temporal lobe. (D) Region of interest analyses. Box plot diagrams with overlaid swarm plots. Shown are the median values as well as within and between‐group variability. *p*‐values (uncorrected) correspond to Mann–Whitney*‐U*‐test. FLE, frontal lobe epilepsy; HC, healthy controls.

Figure 4 and Table S4 depict the group comparisons of normal‐ versus low‐performing FLE patients versus controls.

For face encoding, patients with normal face recognition exhibited lower right mTL activation than controls. For scene encoding and in tendency also for word encoding, only patients with impaired recognition accuracy had less mTL, occipital and (inferior) frontal ROI activation than controls. Those were right‐sided for scenes and left‐sided for words. Similarly, when phenotyping according to delayed verbal recall, impaired patients tended to have less activation in the right orbitofrontal ROI during scene encoding.

When phenotyping according to delayed verbal recall, normal compared to low performing patients, exhibited higher left frontal ROI activation during word encoding and higher right orbitofrontal ROI activation during scene encoding. Likewise, when phenotyping using the respective recognition accuracy, they had increased activation in the left and in tendency right frontal ROI during word and in tendency also in the right inferior frontal ROI during scene encoding. During word encoding normal performing patients further exhibited stronger right mTL ROI, right basal ganglia and left occipital activation and during scene encoding stronger right mTL and in tendency occipital activation.

Finally, in some regions, normal performing patients tended to show more activation than controls. For scene encoding and scene recognition, this increase encompassed left occipital regions. For word encoding and word recognition, increases were found in the right amygdala, which replicated when phenotyping according to delayed verbal recall, and the right frontal ROI.

We further explored which demographical and clinical characteristics in FLE patients might impact memory performance. Therefore, we compared normal and low performing groups regarding these features (see Table S7), which indicated that patients performing low in scene recognition had earlier epilepsy onset and longer epilepsy duration and in tendency a higher drug load.

### 3.6 Correlation analyses

We also calculated correlations of fMRI activation and memory scores for FLE patients and controls (see Supplementary Information IV in the Appendix S1).

In FLE patients, bilaterally increased scene encoding activation in mesial temporal, lateral temporal, basal ganglia, parietal and occipital regions was associated with better scene recognition. Correlations in the word condition were less widespread, more left‐lateralized, and additionally included frontal regions. Correlations encompassed predominantly bilateral frontal regions and basal ganglia, and left lateral temporal, parietal and occipital regions. At a lower threshold, correlations were more bilateral and partly extended into hippocampus and amygdala (*p*_(FWE)_ ≤ .1). Furthermore, verbal learning scores positively correlated with scene encoding activation in predominantly right frontal regions. We found no significant correlation for face encoding, which might be due to the reduced variance with predominantly low memory performance for faces.

## 4 DISCUSSION

This study compared the memory performance, mTL volumes and fMRI encoding activation in predominantly lesional presurgical FLE patients and controls. We used various materials and related memory performance to mTL structures and to fMRI activity during encoding. Results indicated that at least pharmacoresistant FLE affects memory performance and the functional and structural integrity of the mTL, besides causing functional changes in the FL itself.

### 4.1 Behavioral results

FLE patients had worse verbal and nonverbal memory performance than controls. This is in line with recent literature on memory impairment in FLE patients.^3^, ^8^, ^37^, ^38^ Further, verbal memory scores suggested that patients are not impaired in verbal learning but have a consolidation or long‐term retrieval deficit. However, the fMRI data indicated further abnormalities during encoding itself (see below). We suppose that memory impairments reflect contributions of the FL to memory as well as rapid seizure propagation from the frontal to the temporal lobe.^39^, ^40^ In a similar vein, TLE patients have been reported to have executive impairments due to seizure propagation which improve after epilepsy surgery.^41^ As expected and supported by previous studies,^8^, ^37^, ^42^ we did not find behavioral effects of FLE lateralization, although interpretation is limited by the small and unequal sub‐samples. Notably, verbal memory deficits were consistently observed although our sample consisted mainly of rFLE. This accords with FLE resulting in network disruptions rather than disruptions of only focal brain regions.

### 4.2 Structural mTL changes

Left hippocampal volumes were generally larger in FLE patients than in controls. Centeno et al.^9^ also reported increased mTL volumes in FLE patients, namely in the piriform cortex, amygdalae and parahippocampal gyrus bilaterally, but not in the hippocampus. Other studies did not find abnormal mTL volumes in FLE^8^, ^12^, ^13^ which might be due to specific sample characteristics. Given that our patients were recruited during presurgical evaluation and mostly had established lesions, they might have been more severely ill and in some respects more homogeneous than other samples. The neurobiological cause of volume increases is still debated. They could reflect diminished gray and white matter demarcation due to nonatrophic histopathological abnormalities.^43^ Volume increases have also been linked to reorganization.^44^ However, the hippocampal volume increase in FLE seemed to be functionally irrelevant or even dysfunctional according to correlational analysis, whereas in controls larger volumes were correlated with better performance. A similar finding exists for children with FLE.^45^ Here, higher right amygdala, cuneus and occipital cortex volumes were reported in children with impaired compared to unimpaired cognitive performance. These volume increases were suggested to reflect disturbed maturation or inefficient compensatory mechanisms in terms of synaptic sprouting secondary to reduced functional inputs to these brain regions.

### 4.3 Neural correlates of encoding

Encoding activations in FLE patients and controls were widespread. Descriptively, FLE patients exhibited lower frontal activation in all three conditions than controls. Although this observation contradicts a previous finding,^8^ it is rather intuitive that the FL is less activated by cognitive tasks in patients with epileptic activity and structural lesions in the FL. This systemic impact of FLE is also found in other fMRI‐tasks assessing executive functions^46^ or emotion regulation.^47^ In our sample, 91.7% of patients had structural lesions whereas this was the case in only 25% in Centeno et al.,^8^ which might account for the more severe disruption of task‐induced FL recruitment in our patients. Additionally, here, more patients (up to 46%, depending on the material) had impaired memory compared with 22% in Centeno et al.^8^

Group comparisons revealed significant encoding‐related differences primarily in the mTL. During face encoding FLE patients had less activation in the right anterior mTL, for which explorative analysis of sub‐samples suggests it might be driven by rFLE patients. Centeno and colleagues did not report generally reduced mTL activation, at least when comparing mostly nonlesional patients and controls and collapsing across various encoding materials. Instead, a reduction occurred only in their group of low performing patients. Hence, reduced mTL activation might be more common in patients with known structural lesions. It might also be driven by rFLE patients and the face encoding task as previous studies indicated that the right FL and mTL are typically more activated than the left during face encoding.^8^, ^17^, ^33^ Further, inspection of t‐values in controls indicated that both right anterior mTL and right FL activation were stronger during face than during scene or word encoding. Because stronger activation generally increases the possibility to identify between‐group differences, this might have facilitated finding group differences in these regions during face encoding. For face recognition, the comparison of normal and low performing FLE patients revealed no difference in mTL or FL activation. However, due to the low variance of face recognition accuracy, the division of normal and low performers could have been less valid than for other materials. When instead splitting the groups according to median split, ROI analysis showed reduced right mTL and in tendency also right frontal activation only in low performing FLE patients compared to controls.

For scene and for word encoding, we found reduced mTL and FL activation in low performing FLE patients compared with normal performing FLE patients and partly also compared with controls. Clinically, impaired scene recognition was associated with earlier epilepsy onset and longer epilepsy duration. There were hints towards compensatory activation, too: correlation analysis associated higher verbal memory with increased right frontal activation only in FLE patients but not in controls. Reduced mTL activation in low performing FLE patients is in line with previous across‐material results.^8^ For normal performing patients, Centeno et al. reported bilaterally increased FL activation. Our analysis revealed a more specific pattern: In particular, word processing was more bilateral in our normal performing FLE patients, which might reflect reorganization. Hence, memory impairment in FLE seems due to functional changes in the mTL besides the influence of the FL itself.

During word encoding, FLE patients had higher activation than controls especially in the posterior cingulate gyrus. As the overall contrast showed less activation during encoding than during baseline in this region, these activation increases in patients might reflect less deactivation compared with controls. In our previous study TLE patients also exhibited less deactivation than controls in regions which are part of the default mode network.^17^ This finding is reminiscent of an fMRI study on expressive language and working memory.^46^ The study reported for FLE and TLE patients alike reduced deactivation in the default mode network. Together, recent findings indicate that less deactivation in regions, which are part of the default mode network, might be common to different types of focal epilepsies across various tasks.

### 4.4 Strengths and limitations

To the best of our knowledge, our study is the first one comparing material‐specific encoding activation between FLE patients and controls. All patients were undergoing presurgical evaluation and the vast majority had a specific FL lesion. This increases the specificity of the findings, but may reduce the generalizability across FLE. Although we analyzed a sample size within the range of similar studies, we could address effects of FLE lateralization only exploratively and could not distinguish between different FL localizations. Also, the impact of clinical and demographical characteristics (e.g. etiology, language lateralization, antiseizure medication, education) could only be addressed in a preliminary way. Therefore, future studies should extend and confirm our results in larger samples.

## 5 FUTURE DIRECTIONS

Our study underlines the material‐specific impact of FLE on core mTL memory regions, besides the FL itself. FLE patients’ memory impairment might originate not only from frontal changes but also from functional and possibly even structural changes in the mTL, underscoring the functional importance of bidirectionally connected fronto‐temporal networks. Present results are most representative of presurgical FLE patients. Future work will determine whether they generalize to less chronic patients, are reversible by lesion resection, and specify effects of lesion laterality or locus on functional and structural changes. Further, comparison with data from other epilepsy types will help delineate the specificity of results and gauge effects of antiseizure medication. Comparison with our previous study^17^ indicates less severe memory deficits in FLE than TLE and somewhat different underlying activation patterns. Due to space limitations, the direct comparison will be presented elsewhere.

## CONFLICT OF INTEREST STATEMENT

None of the authors has any conflict of interest to disclose.

## ETHICS STATEMENT

We confirm that we have read the journal's position on issues involved in ethical publication and affirm that this report is consistent with those guidelines.

## Supporting information


Appendix S1

## Data Availability

All unthresholded statistical fMRI maps are available on NeuroVault (https://identifiers.org/neurovault.collection:14528). Additional data from this study are available from the corresponding author upon reasonable request.

## References

[1] Lamberink HJ , Otte WM , Blümcke I , Braun KPJ , European Epilepsy Brain Bank writing group , Study Group , et al. Seizure outcome and use of antiepileptic drugs after epilepsy surgery according to histopathological diagnosis: a retrospective multicentre cohort study. Lancet Neurol. 2020;19(9):748–757. 32822635 10.1016/S1474-4422(20)30220-9

[2] Centeno M , Thompson PJ , Koepp MJ , Helmstaedter C , Duncan JS . Memory in frontal lobe epilepsy. Epilepsy Res. 2010;91(2–3):123–132. 20800996 10.1016/j.eplepsyres.2010.07.017

[3] Bremm FJ , Hendriks MPH , Bien CG , Grewe P . Pre‐ and postoperative verbal memory and executive functioning in frontal versus temporal lobe epilepsy. Epilepsy & Behavior: E&B. 2019;101:106538. 10.1016/j.yebeh.2019.106538 31678807

[4] Jeong W , Chung CK , Kim JS . Episodic memory in aspects of large‐scale brain networks. Front Hum Neurosci. 2015;9:454. 26321939 10.3389/fnhum.2015.00454 PMC4536379

[5] Spaniol J , Davidson PS , Kim AS , Han H , Moscovitch M , Grady CL . Event‐related fMRI studies of episodic encoding and retrieval: meta‐analyses using activation likelihood estimation. Neuropsychologia. 2009;47(8–9):1765–1779. 19428409 10.1016/j.neuropsychologia.2009.02.028

[6] Buckner RL , Kelley WM , Petersen SE . Frontal cortex contributes to human memory formation. Nat Neurosci. 1999;2(4):311–314. 10204536 10.1038/7221

[7] Harrington GS , Tomaszewski Farias S , Buonocore MH , Yonelinas AP . The intersubject and intrasubject reproducibility of FMRI activation during three encoding tasks: implications for clinical applications. Neuroradiology. 2006;48(7):495–505. 16703360 10.1007/s00234-006-0083-2

[8] Centeno M , Vollmar C , O'Muircheartaigh J , Stretton J , Bonelli SB , Symms MR , et al. Memory in frontal lobe epilepsy: an fMRI study. Epilepsia. 2012;53(10):1756–1764. 22765637 10.1111/j.1528-1167.2012.03570.x PMC7116587

[9] Centeno M , Vollmar C , Stretton J , Symms MR , Thompson PJ , Richardson MP , et al. Structural changes in the temporal lobe and piriform cortex in frontal lobe epilepsy. Epilepsy Res. 2014;108(5):978–981. 24726451 10.1016/j.eplepsyres.2014.03.001 PMC4037873

[10] Braakman HMH , Vaessen MJ , Jansen JFA , Debeij‐van Hall MHJA , de Louw A , Hofman PAM , et al. Pediatric frontal lobe epilepsy: white matter abnormalities and cognitive impairment. Acta Neurol Scand. 2014;129(4):252–262. 24112290 10.1111/ane.12183

[11] Widjaja E , Mahmoodabadi SZ , Snead OC , Almehdar A , Smith ML . Widespread cortical thinning in children with frontal lobe epilepsy. Epilepsia. 2011;52(9):1685–1691. 21627647 10.1111/j.1528-1167.2011.03085.x

[12] Vu L , Swartz B , Head K , Mandelkern M , Nalcioglu O , Su MY . Voxel based morphometry and manual volumetric analysis of the cingulate gyrus in patients with juvenile myoclonic epilepsy and frontal lobe epilepsy compared to normal controls. Proc Intl Soc Mag Reson Med. 2006;14:3123.

[13] Lu CQ , Gosden GP , Okromelidze L , Jain A , Gupta V , Grewal SS , et al. Brain structural differences in temporal lobe and frontal lobe epilepsy patients: a voxel‐based morphometry and vertex‐based surface analysis. Neuroradiol J. 2022;35(2):193–202. 34313179 10.1177/19714009211034839 PMC9130607

[14] Bonelli SB , Powell RH , Yogarajah M , Samson RS , Symms MR , Thompson PJ , et al. Imaging memory in temporal lobe epilepsy: predicting the effects of temporal lobe resection. Brain. 2010;133(4):1186–1199. 20157009 10.1093/brain/awq006 PMC2850579

[15] Sidhu MK , Stretton J , Winston GP , Symms M , Thompson PJ , Koepp MJ , et al. Memory fMRI predicts verbal memory decline after anterior temporal lobe resection. Neurology. 2015;84(15):1512–1519. 25770199 10.1212/WNL.0000000000001461 PMC4408284

[16] Richardson MP , Strange BA , Thompson PJ , Baxendale SA , Duncan JS , Dolan RJ . Pre‐operative verbal memory fMRI predicts post‐operative memory decline after left temporal lobe resection. Brain. 2004;127(11):2419–2426. 15459025 10.1093/brain/awh293

[17] Doll A , Wegrzyn M , Benzait A , Mertens M , Woermann FG , Labudda K , et al. Whole‐brain functional correlates of memory formation in mesial temporal lobe epilepsy. Neuroimage Clin. 2021;31:102723. 34147817 10.1016/j.nicl.2021.102723 PMC8220377

[18] Woermann FG , Jokeit H , Luerding R , Freitag H , Schulz R , Guertler S , et al. Language lateralization by Wada test and fMRI in 100 patients with epilepsy. Neurology. 2003;61(5):699–701. 12963768 10.1212/01.wnl.0000078815.03224.57

[19] Engel J Jr , Van Ness PC , Rasmussen TB , Ojemann LM . Outcome with respect to epileptic seizures. In: Engel J Jr , editor. Surgical treatment of the epilepsies. New York: Raven Press; 1993. p. 609–621.

[20] Schmidt M . Rey auditory verbal learning test: a handbook. Los Angeles, CA: Western Psychological Services; 1996.

[21] Helmstaedter C , Lendt M , Lux S . Verbaler Lern‐ und Merkfähigkeitstest: VLMT; Manual. Göttingen: Beltz Test; 2001.

[22] Weidlich S , Derouiche A , Hartje W . Diagnosticum für Cerebralschädigung ‐ II: DCS‐II; ein figuraler Lern‐ und Gedächtnistest; Manual. Bern: Huber Hogrefe; 2011.

[23] Esteban O , Markiewicz CJ , Blair RW , Moodie CA , Isik AI , Erramuzpe A , et al. fMRIPrep: a robust preprocessing pipeline for functional MRI. Nat Methods. 2019;16(1):111–116. 30532080 10.1038/s41592-018-0235-4 PMC6319393

[24] Snodgrass JG , Corwin J . Pragmatics of measuring recognition memory: applications to dementia and amnesia. J Exp Psychol Gen. 1988;117(1):34–50. 2966230 10.1037//0096-3445.117.1.34

[25] Rubin M . When to adjust alpha during multiple testing: a consideration of disjunction, conjunction, and individual testing. Synthese. 2021;199(3–4):10969–11000.

[26] Althouse AD . Adjust for multiple comparisons? It's not that simple. Ann Thorac Surg. 2016;101(5):1644–1645. 27106412 10.1016/j.athoracsur.2015.11.024

[27] Fischl B , Salat DH , Busa E , Albert M , Dieterich M , Haselgrove C , et al. Whole brain segmentation: automated labeling of neuroanatomical structures in the human brain. Neuron. 2002;33(3):341–355. 11832223 10.1016/s0896-6273(02)00569-x

[28] Fischl B , van der Kouwe A , Destrieux C , Halgren E , Ségonne F , Salat DH , et al. Automatically parcellating the human cerebral cortex. Cereb Cortex. 2004;14(1):11–22. 14654453 10.1093/cercor/bhg087

[29] Winkler AM , Ridgway GR , Webster MA , Smith SM , Nichols TE . Permutation inference for the general linear model. Neuroimage. 2014;92:381–397. 24530839 10.1016/j.neuroimage.2014.01.060 PMC4010955

[30] Smith N , Nichols TE . Threshold‐free cluster enhancement: addressing problems of smoothing, threshold dependence and localisation in cluster inference. Neuroimage. 2009;44(1):83–98. 18501637 10.1016/j.neuroimage.2008.03.061

[31] Bennett CM , Wolford GL , Miller MB . The principled control of false positives in neuroimaging. Soc Cogn Affect Neurosci. 2009;4(4):417–422. 20042432 10.1093/scan/nsp053 PMC2799957

[32] Roiser JP , Linden DE , Gorno‐Tempinin ML , Moran RJ , Dickerson BC , Grafton ST . Minimum statistical standards for submissions to Neuroimage: clinical. Neuroimage Clin. 2016;12:1045–1047. 27995071 10.1016/j.nicl.2016.08.002 PMC5153601

[33] Powell HW , Koepp MJ , Symms MR , Boulby PA , Salek‐Haddadi A , Thompson PJ , et al. Material‐specific lateralization of memory encoding in the medial temporal lobe: blocked versus event‐related design. Neuroimage. 2005;27(1):231–239. 15927485 10.1016/j.neuroimage.2005.04.033

[34] Strange BA , Otten LJ , Josephs O , Rugg MD , Dolan RJ . Dissociable human perirhinal, hippocampal, and parahippocampal roles during verbal encoding. J Neurosci. 2002;22(2):523–528. 11784798 10.1523/JNEUROSCI.22-02-00523.2002 PMC6758661

[35] Olman CA , Davachi L , Inati S . Distortion and signal loss in medial temporal lobe. PloS One. 2009;4(12):e8160. 19997633 10.1371/journal.pone.0008160 PMC2780716

[36] Yarkoni T , Poldrack R , Nichols T , Van Essen D , Wagner T . NeuroSynth: a new platform for large‐scale automated synthesis of human functional neuroimaging data. Front. Neuroinform. Conference Abstract: 4th INCF Congress of Neuroinformatics. 2011. 10.1038/nmeth.1635 PMC3146590 21706013

[37] Exner C , Boucsein K , Lange C , Winter H , Weniger G , Steinhoff BJ , et al. Neuropsychological performance in frontal lobe epilepsy. Seizure. 2002;11(1):20–32. 11888256 10.1053/seiz.2001.0572

[38] Cahn‐Weiner DA , Wittenberg D , McDonald C . Everyday cognition in temporal lobe and frontal lobe epilepsy. Epileptic Disord. 2009;11(3):222–227. 19713170 10.1684/epd.2009.0265

[39] Kibby MY , Cohen MJ , Stanford L , Park YD . Are frontal and temporal lobe epilepsy dissociable in their memory functioning? Epilepsy Behav. 2019;99:106487. 31476730 10.1016/j.yebeh.2019.106487

[40] Bagla R , Skidmore CT . Frontal lobe seizures. Neurologist. 2011;17(3):125–135. 21532379 10.1097/NRL.0b013e31821733db

[41] Nair S , Szaflarski JP . Neuroimaging of memory in frontal lobe epilepsy. Epilepsy Behav. 2020;103(Pt A):106857. 31937510 10.1016/j.yebeh.2019.106857

[42] Busch RM , Floden DP , Ferguson L , Mahmoud S , Mullane A , Jones S , et al. Neuropsychological outcome following frontal lobectomy for pharmacoresistant epilepsy in adults. Neurology. 2017;88(7):692–700. 28087827 10.1212/WNL.0000000000003611 PMC5317375

[43] Woermann FG , Free SL , Koepp MJ , Ashburner J , Duncan JS . Voxel‐by‐voxel comparison of automatically segmented cerebral gray matter—a rater‐independent comparison of structural MRI in patients with epilepsy. Neuroimage. 1999;10(4):373–384. 10493896 10.1006/nimg.1999.0481

[44] Labudda K , Mertens M , Janszky J , Bien CG , Woermann FG . Atypical language lateralisation associated with right fronto‐temporal grey matter increases — a combined fMRI and VBM study in left‐sided mesial temporal lobe epilepsy patients. Neuroimage. 2012;59(1):728–737. 21839176 10.1016/j.neuroimage.2011.07.053

[45] Braakman HM , Vaessen MJ , Jansen JF , Debeij‐van Hall MH , de Louw A , Hofman PA , et al. Aetiology of cognitive impairment in children with frontal lobe epilepsy. Acta Neurol Scand. 2015;131(1):17–29. 25208759 10.1111/ane.12283

[46] Caciagli L , Paquola C , He X , Vollmar C , Centeno M , Wandschneider B , et al. Disorganization of language and working memory systems in frontal versus temporal lobe epilepsy. Brain. 2023;146(3):935–953. 35511160 10.1093/brain/awac150 PMC9976988

[47] Benzait A , Krenz V , Wegrzyn M , Doll A , Woermann F , Labudda K , et al. Hemodynamic correlates of emotion regulation in frontal lobe epilepsy patients and healthy participants. Hum Brain Mapp. 2023;44(4):1456–1475. 36366744 10.1002/hbm.26133 PMC9921231

