# Supplementary material for: MRI evidence for material‐specific encoding deficits and mesial–temporal alterations in presurgical frontal lobe epilepsy patients
Source: Epilepsia Open. 2024 Jan 4;9(1):355–67. doi: 10.1002/epi4.12881 (PMC10839294; doi:10.1002/epi4.12881)
Supplement: Supplementary file 1 — Appendix S1 [file EPI4-9-355-s001.docx]

**Supporting Information**

**Supplementary Information I**

Table S1

*List of stimuli used in the experiment*

| pictures | negative | neutral |
| --- | --- | --- |
| IAPS pictures^1^ | 1120  1205  1275  1300  1304  1932  2095  2205  2276  2683  2688  2710  2800  2811  2981  3103  3181  3185  3213  3230  3300  3301  3350  3550  6021  6311  6313  6415  6520  6821  6834  7380  8230  8485  9000  9006  9007  9040  9041  9043  9140  9265  9402  9421  9432  9433  9480  9560  9584  9611  9630  9800  9810  9909  9940 | 1450  1675  1910  2026  2038  2102  2308  2372  2377  2384  2390  2393  2396  2397  2400  2411  2435  2440  2484  2487  2495  2499  2513  2521  2525  2575  2579  2580  2594  2635  2745  2749  2840  2850  5471  5531  7009  7187  7476  7491  7512  7513  7550  7632  8241  8312 |
| own dataset | child soldier  burning house  mouldy orange  mouldy toast  bloody face (mini-3051) | wooden chair  empty dental practice  people sitting at a table  car  church building (N77A)  hiking shoes and socks  train  crowned crane  mallard duck  pot-bellied pig  fox  toad  bird  person cooking a meal |
| faces | negative | neutral |
| NimStim faces^2^ | female  01F_FE_O  02F_FE_O  03F_FE_O  05F_FE_O  06F_FE_O  07F_FE_O  08F_FE_O  09F_FE_O  10F_FE_O  11F_FE_O  17F_FE_O  18F_FE_O  male  20M_FE_O  21M_FE_O  22M_FE_O  23M_FE_O  24M_FE_O  25M_FE_O  27M_FE_O  28M_FE_O  29M_FE_O  30M_FE_O  36M_FE_O  37M_FE_O | female  01F_NE_C  02F_NE_C  03F_NE_C  05F_NE_C  06F_NE_C  07F_NE_C  08F_NE_C  09F_NE_C  10F_NE_C  11F_NE_C  17F_NE_C  18F_NE_C  male  20M_NE_C  21M_NE_C  22M_NE_C  23M_NE_C  24M_NE_C  25M_NE_C  27M_NE_C  28M_NE_C  29M_NE_C  30M_NE_C  36M_NE_C  37M_NE_C |
| FACES database^3^ | female - young age  010_y_f_f_b  020_y_f_f_a  022_y_f_f_a  040_y_f_f_b  048_y_f_f_b  054_y_f_f_a  098_y_f_f_a  101_y_f_f_b  106_y_f_f_b  115_y_f_f_a  163_y_f_f_b  182_y_f_f_b  male - young age  008_y_m_f_b  013_y_m_f_a  041_y_m_f_b  057_y_m_f_a  066_y_m_f_a  072_y_m_f_a  089_y_m_f_a  109_y_m_f_b  114_y_m_f_a  123_y_m_f_a  167_y_m_f_a  170_y_m_f_b  female - middle age  006_m_f_f_b  011_m_f_f_b  019_m_f_f_a  029_m_f_f_a  035_m_f_f_b  050_m_f_f_a  052_m_f_f_b  061_m_f_f_a  064_m_f_f_b  073_m_f_f_a  080_m_f_f_b  084_m_f_f_b  male - middle age  007_m_m_f_a  014_m_m_f_a  026_m_m_f_a  032_m_m_f_b  038_m_m_f_b  045_m_m_f_a  056_m_m_f_a  058_m_m_f_b  068_m_m_f_b  070_m_m_f_a  077_m_m_f_b  082_m_m_f_a  female - old age  005_o_f_f_a  012_o_f_f_b  021_o_f_f_b  024_o_f_f_b  036_o_f_f_b  044_o_f_f_a  047_o_f_f_a  055_o_f_f_b  060_o_f_f_a  067_o_f_f_a  075_o_f_f_a  079_o_f_f_b  male - old age  004_o_m_f_a  015_o_m_f_b  018_o_m_f_b  027_o_m_f_b  039_o_m_f_b  042_o_m_f_a  046_o_m_f_a  053_o_m_f_a  059_o_m_f_a  065_o_m_f_a  074_o_m_f_b  076_o_m_f_a | female - young age  010_y_f_n_a  020_y_f_n_a  022_y_f_n_a  040_y_f_n_a  048_y_f_n_a  054_y_f_n_a  098_y_f_n_a  101_y_f_n_a  106_y_f_n_a  115_y_f_n_a  163_y_f_n_a  182_y_f_n_a  male - young age  008_y_m_n_a  013_y_m_n_a  041_y_m_n_a  057_y_m_n_a  066_y_m_n_a  072_y_m_n_a  089_y_m_n_a  109_y_m_n_a  114_y_m_n_a  123_y_m_n_a  167_y_m_n_a  170_y_m_n_a  female - middle age  006_m_f_n_a  011_m_f_n_b  019_m_f_n_b  029_m_f_n_a  035_m_f_n_a  050_m_f_n_b  052_m_f_n_a  061_m_f_n_a  064_m_f_n_b  073_m_f_n_b  080_m_f_n_a  084_m_f_n_b  male - middle age  007_m_m_n_a  014_m_m_n_a  026_m_m_n_b  032_m_m_n_a  038_m_m_n_a  045_m_m_n_b  056_m_m_n_a  058_m_m_n_a  068_m_m_n_b  070_m_m_n_b  077_m_m_n_b  082_m_m_n_b  female - old age  005_o_f_n_b  012_o_f_n_b  021_o_f_n_a  024_o_f_n_a  036_o_f_n_a  044_o_f_n_a  047_o_f_n_a  055_o_f_n_b  060_o_f_n_a  067_o_f_n_b  075_o_f_n_a  079_o_f_n_b  male - old age  004_o_m_n_b  015_o_m_n_a  018_o_m_n_a  027_o_m_n_a  039_o_m_n_b  042_o_m_n_a  046_o_m_n_a  053_o_m_n_b  059_o_m_n_b  065_o_m_n_b  074_o_m_n_b  076_o_m_n_b |
| KDEF^4^ | female  AF01AFS  AF06AFS  AF07AFS  AF11AFS  AF13AFS  AF14AFS  AF15AFS  AF16AFS  AF18AFS  AF19AFS  AF30AFS  AF31AFS  male  AM01AFS  AM02AFS  AM04AFS  AM05AFS  AM06AFS  AM07AFS  AM08AFS  AM10AFS  AM11AFS  AM13AFS  AM22AFS  AM23AFS | female  AF01NES  AF06NES  AF07NES  AF11NES  AF13NES  AF14NES  AF15NES  AF16NES  AF18NES  AF19NES  AF30NES  AF31NES  male  AM01NES  AM02NES  AM04NES  AM05NES  AM06NES  AM07NES  AM08NES  AM10NES  AM11NES  AM13NES  AM22NES  AM23NES |
| words | negative | neutral |
| own dataset^5^ | Alptraum (nightmare)  Angst (fear)  Beklemmung (anxiety)  Bestie (beast)  Blamage (disgrace)  Blut (blood)  Brutalität (brutality)  Demütigung (humiliation)  Diebstahl (robbery)  Diktator (dictator)  Durchfall (diarrhoea)  Eifersucht (jealousy)  Eiter (pus)  Ekel (disgust)  Ekzem (eczema)  Elend (misery)  Erpresser (blackmailer)  Explosion (explosion)  Fixer (junkie)  Fluch (curse)  Folter (torture)  Geisel (hostage)  Geschwür (ulcer)  Habgier (greed)  Henker (executioner)  Heroin (heroin)  Hetze (rabble-rousing)  Hilflosigkeit (helplessness)  Hunger (hunger)  Hungersnot (famine)  Isolation (isolation)  Kälte (cold)  Katastrophe (catastrophe)  Kerker (dungeon)  Kreuzigung (crucifixion)  Leiden (suffering)  Lepra (leprosy)  Lügner (liar)  Lungenkrebs (lung cancer)  Narbe (scar)  Nazi (Nazi)  Opfer (victim)  Panik (panic)  Perversion (perversion)  Pisse (piss)  Rassismus (racism)  Selbstmord (suicide)  Seuche (epidemic)  Sklaverei (slavery)  Spritze (syringe)  Teufel (devil)  Tumor (tumor)  Ungerechtigkeit (injustice)  Untergang (doom)  Vergewaltigung (rape)  Verrat (betrayal)  Verstümmelung (mutilation)  Wahn (delusion)  Warze (wart)  Wunde (wound) | Aktentasche (briefcase)  Armbeuge (arm bend)  Automat (automat)  Batterie (battery)  Beleg (receipt)  Bewohner (inhabitant)  Biegung (bend)  Bleistift (pencil)  Brause (sherbet)  Bügeleisen (flat iron)  Computer (computer)  Detail (detail)  Eigenschaft (characteristic)  Fahrkarte (ticket)  Faktor (factor)  Flasche (bottle)  Flugzeug (aircraft)  Gerüst (scaffold)  Geschirr (dishes)  Getreide (grain)  Hausschuhe (slippers)  Information (information)  Kanister (canister)  Kastanie (chestnut)  Kasten (box)  Kleiderbügel (clothes hanger)  Klingel (bell)  Kran (crane)  Kurve (curve)  Merkmal (feature)  Mikroskop (microscope)  Motorrad (motorcycle)  Natrium (sodium)  Objekt (object)  Papier (paper)  Partikel (particle)  Plastik (plastic)  Post (post)  Pronomen (pronoun)  Quadrat (square)  Rasen (lawn)  Reflex (reflex)  Regal (shelf)  Reifen (tire)  Rolltreppe (escalator)  Ruder (rudder)  Sicht (view)  Siedlung (settlement)  Spiegel (mirror)  Stellvertretung (representation)  Symbol (symbol)  Tablett (tray)  Tastatur (keyboard)  Truhe (chest)  Turban (turban)  Ufer (shore)  Unterlage (base)  Vorhang (curtain)  Votum (vote)  Ziegel (brick) |

1. Lang PJ, Bradley MM, Cuthbert BN. International Affective Picture System (IAPS): affective ratings of pictures and instruction manual. *Technical Report A-8*. 2008.

2. Tottenham N, Tanaka JW, Leon AC*, et al.* The NimStim set of facial expressions: judgments from untrained research participants. *Psychiatry Research*. 2009;168(3):242-249.

3. Ebner NC, Riediger M, Lindenberger U. FACES--a database of facial expressions in young, middle-aged, and older women and men: development and validation. *Behavior Research Methods*. 2010;42(1):351-362.

4. Lundqvist, D., Flykt, A., & Öhman, A. (1998). The Karolinska directed emotional faces (KDEF). CD ROM from Department of Clinical Neuroscience, Psychology section, Karolinska Institutet, 91(630), 2-2.

5. Kissler, J., Herbert, C., Peyk, P., & Junghofer, M. (2007). Buzzwords: early cortical responses to emotional words during reading. *Psychological science*, *18*(6), 475-480.

**Supplementary Information II**

Table S2 *Percentage of hits and false alarms, recognition accuracy and response bias of scenes, faces and words separately for controls and FLE patients.*

|  | Scenes | Faces | | | Words | |
| --- | --- | --- | --- | --- | --- | --- |
|  | Controls | FLE patients | Controls | FLE patients | Controls | FLE patients |
| Hit | 86.1  (62.5; 98.6) | 75.7 *  (33.3; 97.2) | 56.3  (36.1; 79.2) | 52.1  (27.8; 83.3) | 77.1 *  (48.6; 95.8) | 68.1  (36.1; 90.3) |
|  | *U*=509.5, *p*=.009, *d*=0.76 | | - |  | *U*=460.0, *p*=.02, *d*=0.71 | |
| False alarm | 7.3  (0.0; 62.5) | 4.2  (0.0; 43.8) | 35.4  (0; 64.6) | 36.5  (4.2; 70.8) | 17.7  (4.2; 50.0) | 24.0  (8.3; 58.3) |
| Recognition accuracy | 79.5  (31.9; 96.5) | 68.8  (27.1; 93.1) | 26.0  (-4.9; 46.5) | 16.3 ^#^  (-16.0; 45.8) | 55.2 *  (29.2; 83.3) | 39.9  (12.5; 68.7) |
|  | - |  | *U*=462.5, *p*=.08, *d*=0.50 | | *U*=490.5, *p*=.003, *d*=0.91 | |
| Response bias | 0.38  (0.0; 0.96) | 0.22  (0.0; 0.62) | 0.45  (0.0; 0.76) | 0.45  (0.06; 0.81) | 0.44  (0.10; 0.91) | 0.42  (0.12; 0.75) |

*Note.* Percentage [*Mdn* (range)].* *p* ≤ .05 and ^#^ *p* ≤ .1 according to the Mann-Whitney *U* Test used to compare controls with FLE patients. Abbreviations: FLE, frontal lobe epilepsy.

Table S3 *Verbal learning, delayed verbal recall and design learning scores separately for controls and FLE patients.*

|  | Verbal learning | | Delayed verbal recall | | Design Leaning | |
| --- | --- | --- | --- | --- | --- | --- |
|  | Controls | FLE patients | Controls | FLE patients | Controls | FLE patients |
| Scores | 0.76  (-1.28; 1.96) | 0.52  (-1.04; 1.96) | 0.93  (-1.2; -1.95) | 0.13*  (-1.96; -1.64) | 0.68,  (-1.0; -2.0) | 0.07*  (-1.61; -1.72) |
|  | *-* | | *U*=485.5, *p*=.03, *d*=0.62 | | *U*=478.0, *p*=.04, *d*=0.58 | |

*Note.* Percentage [*Mdn* (range)].* *p* ≤ .05 and ^#^ *p* ≤ .1 according to the Mann-Whitney *U* Test used to compare controls with FLE patients. Abbreviations: FLE, frontal lobe epilepsy.

**Supplementary Information III**

Table S4

*Group comparisons of fMRI activation of controls and* ***A*** *FLE patients and* **B** *normal and low performing FLE patients during scene, face and word encoding.*

| **A. Group comparison: FLE patients vs. controls** | | | | | | | | | | | |
| --- | --- | --- | --- | --- | --- | --- | --- | --- | --- | --- | --- |
|  | Clustersize  [mm³] | | | Peak | *p*_(FWE)_ | | | *t* | Peak Region | Cluster Regions [% of cluster in the respective region] | |
| **Faces** | | | | | | | | | | | |
| **HC > FLE** | 1200* | 32 -5 -23 | | | | | .02 | 3.9 | right amygdala | 60.0% right hippocampus; 39.3% right amygdala | |
| **HC > lFLE** | 7088 | 22 -77 36 | | | | | .03 | 5.1 | right lateral occipital cortex | 27.2% right lateral occipital cortex; 23.6% left lateral occipital cortex; 22.2% right cuneal cortex; 17.0 left cuneal cortex; 4.1% left occipital pole; 3.2% right precuneus cortex | |
|  | 4024 | 20 -65 14 | | | | | .05 | 4.6 | right supracalcarine cortex | 44.3% right lingual gyrus; 15.3% right temporal occipital fusiform cortex; 13.3% right precuneus cortex; 9.3% right cingulate gyrus; 7.8% right supracalcarine cortex; 4.8% right intracalcarine cortex | |
|  | 904 | 8 -65 2 | | | | | .08 | 3.5 | right lingual gyrus | 85.0% right lingual gyrus; 15.0 % right intracalcarine cortex | |
|  | 200 | -15 -91 22 | | | | | .09 | 3.6 | left occipital pole | 88.0% left occipital pole; 8.0% left cuneal cortex | |
| **HC > rFLE** | 1640* | 28 -13 -15 | | | | | .007 | 4.1 | right hippocampus | 68.8% right hippocampus; 31.2% right amygdala | |
| **Words** | | | | | | | | | | | |
| **FLE > HC** | 2264 | -9 -17 34 | | | | | .03 | 5.8 | no label | 69.3% left cingulate gyrus; 25.4% left cingulate gyrus | |
|  | 176 | 54 -21 24 | | | | | .08 | 4.9 | no label | 45.5% right postcentral gyrus; 36.4% right opercular cortex; 13.6% right supramarginal gyrus | |
| **rFLE > HC** | 152 | -9 -19 32 | | | | | .06 | 5.8 | no label | 91.3% left cingulate gyrus | |
|  |  |  | | | | |  |  |  |  | |
| **B. Group comparison: normal vs. low performing FLE patients vs. controls** | | | | | | | | | | |  |
| **Scene encoding and phenotyping using respective recognition accuracy** | | | | | | | | | | |  |
| HC > FLE low | 144 | 50 -81 14 | | | | | .09 | 4.4 | no label | 94.4% right lateral occipital cortex |  |
|  | 416* | 22 -19 -15 | | | | | .02 | 4.3 | right hippocampus | 71.2% right hippocampus; 28.9% right amygdala |  |
| FLE normal > low | 432 | 32 -81 36 | | | | | .08 | 4.3 | right lateral occipital cortex | 100% right lateral occipital cortex |  |
|  | 456* | 22 -21 -15 | | | | | .01 | 4.7 | right hippocampus | 87.7% right hippocampus; 12.3% right amygdala |  |
| FLE normal > HC | 728 | -13 -109 2 | | | | | .06 | 5.7 | no label | 92.2% left occipital pole |  |
| **Face encoding and phenotyping using respective recognition accuracy** | | | | | | | | | | |  |
| HC > FLE normal | 440* | | 18 -13 -15 | | | .04 | | 3.5 | right amygdala | 61.8% right hippocampus; 38.2 % right amygdala |  |
| **Word encoding and phenotyping using respective recognition accuracy** | | | | | | | | | | |  |
| HC > FLE low | 544 | | -23 -73 50 | | | .08 | | 4.7 | left lateral occipital cortex | 100% left lateral occipital cortex |  |
|  | 352 | | -9 -77 62 | | | .07 | | 5.1 | no label | 93.2% left lateral occipital cortex |  |
| FLE normal > low | 1088 | | -25 -79 54 | | | .03 | | 5.7 | no label | 99.3% left lateral occipital cortex |  |
|  | 720 | | 20 -17 22 | | | .06 | | 4.7 | no label | 52.2% right caudate |  |
|  | 288 | | -11 -77 60 | | | .09 | | 4.4 | no label | 97.2% left lateral occipital cortex |  |
| FLE normal > HC | 72* | | 18 -3 -13 | | | .06 | | 4.3 | right amygdala | 100% right amygdala |  |
| **Word encoding and phenotyping using delayed verbal recall scores** | | | | | | | | | | |  |
| FLE normal > HC | 16* | | 20 -3 -13 | | | .09 | | 3.9 | right amygdala | 100% right amygdala |  |

Note. *Small volume correction in the mesial temporal lobe. Abbreviations: FLE, Frontal lobe epilepsy; FWE, family-wise error rate; HC, healthy controls lFLE, left frontal lobe epilepsy; rFLE right frontal lobe epilepsy.

**Supplementary Information IV**

We calculated correlations of fMRI activations and memory scores for controls (see Figure S1) and FLE patients (see Figure S2). In controls increased word encoding activation in left frontal, lateral temporal, parietal, occipital, and in bilateral basal ganglia regions, was associated with higher word recognition accuracy. Activation in largely similar brain regions, additionally including the mesial temporal lobe, was also correlated with verbal learning performance and delayed verbal recall. Correlations in the face condition were very circumscribed. Only increased right hippocampus and amygdala activation correlated with higher recognition accuracy. There was no significant correlation for scene encoding. This might be due to reduced variance in memory performance with mostly very high performance for scenes.


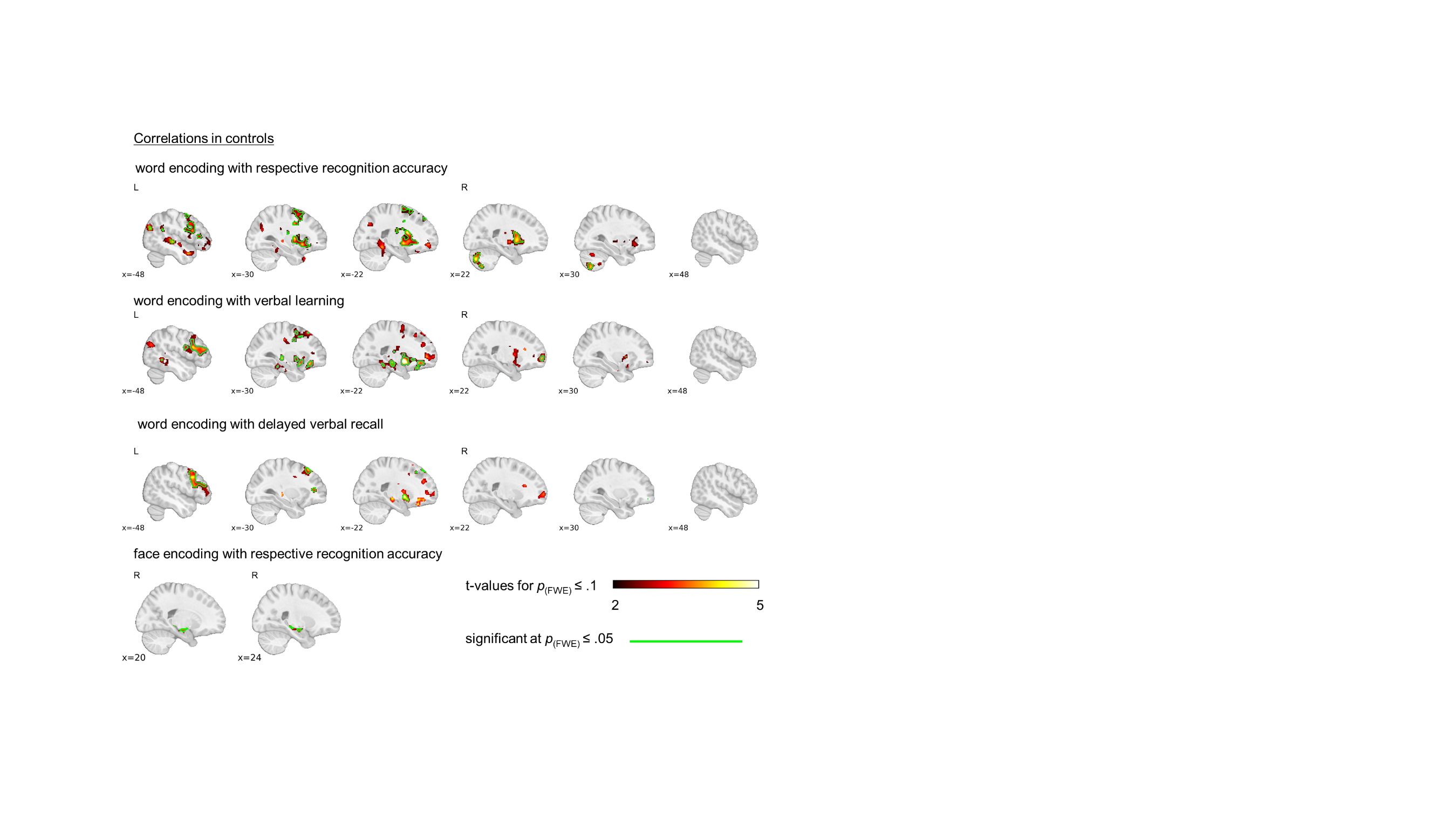


*Figure S1.* **Correlation analysis.** Correlation of word and face encoding activation with the respective recognition accuracy in controls. The colour code indicates t-values significant at *p*_(FWE)_ ≤ .1 and the black outline indicates *p*_(FWE)_ ≤.05, both with SVC for the mesial temporal lobe.

*
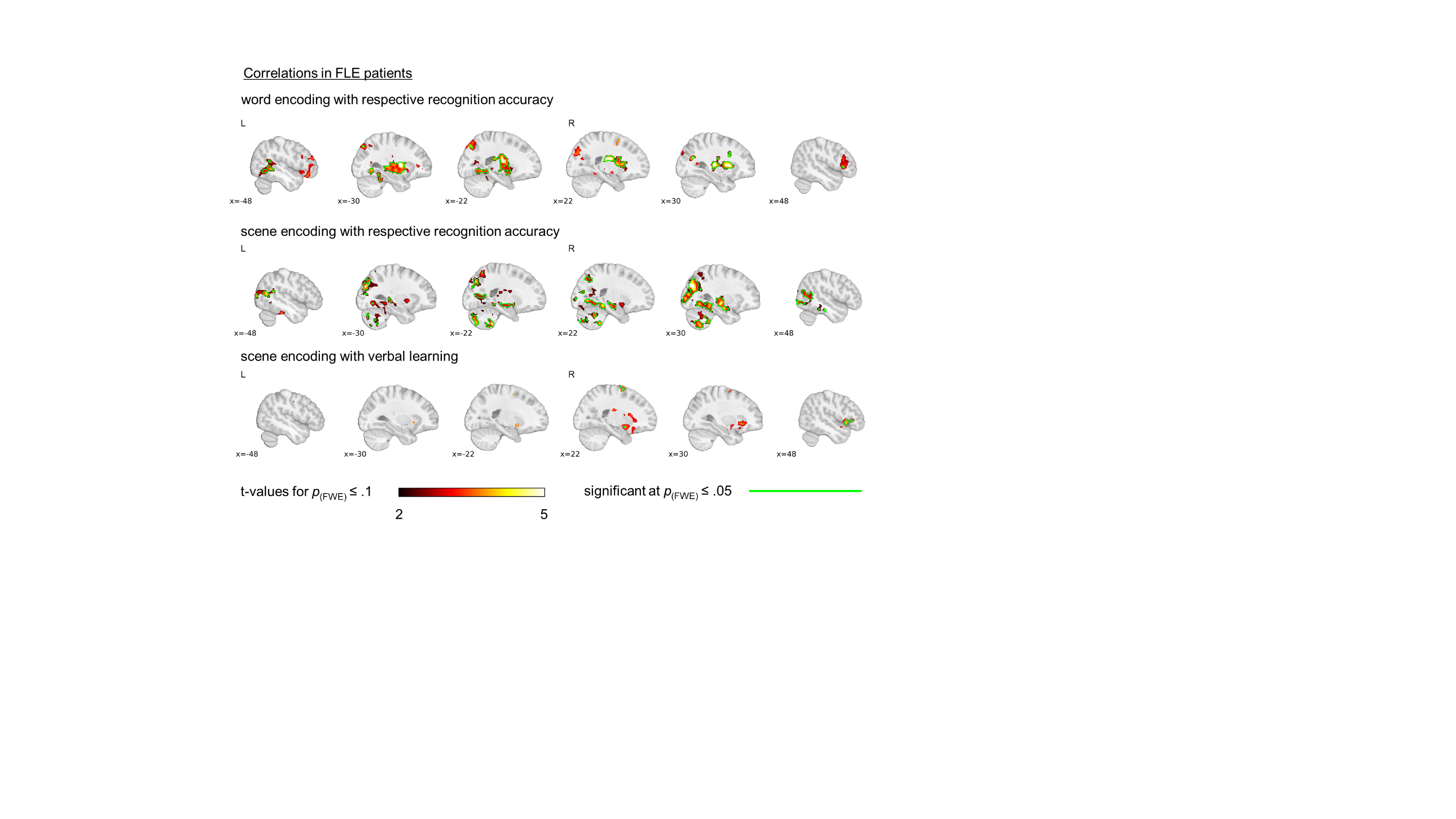
*

*Figure S2.* **Correlation analysis.** Correlation of scene and word encoding activation with memory performance in FLE patients. The colour code indicates t-values significant at *p*_(FWE)_ ≤ .1 and the black outline indicates *p*_(FWE)_ ≤.05, both with SVC for the mesial temporal lobe. Abbreviations: FLE, frontal lobe epilepsy.

Table S5

*Correlation of fMRI activation of FLE patients during scene and word encoding with memory performance.*

| **Correlations in FLE patients: encoding activation with respective recognition accuracies** | | | | | | |
| --- | --- | --- | --- | --- | --- | --- |
|  | Clustersize  [mm³] | Peak | *p*_(FWE)_ | *t* | Peak Region | Cluster Regions [% of cluster in the respective region] |
| **Scene encoding** | | | | | | |
| **scene recogn.** | 153832 | 30 -81 30 | .002 | 6.1 | right lateral occipital cortex | 14.3% right lateral occipital cortex; 10.4% left lateral occipital cortex; 3.7% right lingual gyrus; 3.4% right middle temporal gyrus; 3.4% brain stem; 3.1% right temporal occipital fusiform cortex; 2.6% right occipital fusiform gyrus; 2.4% left intracalcarine cortex; 2.4% right occipital pole; 2.2% left lingual gyrus; 1.8% right hippocampus; 1.4% left occipital fusiform gyrus; 1.3% left temporal fusiform cortex; 1.3% left hippocampus; 1.2% right putamen; 1.2% left thalamus; 1.2% left temporal occipital fusiform cortex; 1.1% right temporal fusiform cortex; 0.8% right thalamus; 0.9% left caudate; 0.9% right amygdala; 0.8% left precuneus cortex; 0.7% right inferior temporal gyrus; 0.7% right intracalcarine cortex; 0.7% left putamen; 0.6% right precuneus cortex; 0.6% left inferior temporal gyrus; 0.6% left angular gyrus; 0.6% left amygdala; 0.6% left middle temporal gyrus; 0.5% right pallidum; 0.4% right parahippocampal gyrus; 0.4%; left parahippocampal gyrus; 0.3% left occipital pole; 0.3% left pallidum; 0.3% right angular gyrus; 0.3% left cingulate gyrus; 0.2% left supramarginal gyrus; 0.2% left inferior temporal gyrus; ; 0.2% right supracalcarine cortex; 0.2% right inferior temporal gyrus; 0.1% left cuneal cortex; 0.1% right superior parietal lobule; 0.1% right cingulate gyrus; 0.06% right caudate |
|  | 1720* | 32 -11 -19 | .008 | 5.2 | right hippocampus | 71.6% right hippocampus; 28.4% right amygdala |
|  | 96* | -25 -15 -13 | .08 | 3.6 | left amygdala | 66.7% left amygdala; 33.3% left hippocampus |
| **verbal learning** | 12280 | 44 16 -3 | .03 | 4.6 | right insular cortex | 24.1% right insular cortex; 19.0% right frontal orbital cortex; 11.3% right frontal operculum cortex; 8.9% right putamen; 7.8% right inferior frontal gyrus pars triangularis; 6.3% right caudate; 4.9% right amygdala; 4.4% right temporal pole; 2.4% right subcallosal cortex; 2.1% right planum polare; 1.9% right central opercular cortex; 1.5% right inferior frontal gyrus pars opercularis; 1.1% right frontal pole |
|  | 2870 | 16 -5 60 | .01 | 7.2 | no label | 90.0% right superior frontal gyrus; 6.4% right juxtapositional lobule; 3.1% right precentral gyrus |
|  | 1360 | 16 -3 18 | .07 | 4.1 | right caudate | 72.9% right caudate; 8.2% right thalamus; 4.1% right putamen |
|  | 976 | -29 16 -5 | .07 | 4.5 | no label | 64.8% left putamen; 16.4% left amygdala; 7.4% left insular cortex; 6.6% left pallidum |
|  | 536 | 36 -5 64 | .06 | 5.0 | right precentral gyrus | 71.6% right precentral gyrus; 19.4% right middle frontal gyrus; 9.9% right superior frontal gyrus |
|  | 80 | -21 -3 58 | .10 | 3.9 | left superior frontal gyrus | 90.0% left superior frontal gyrus; 10.0% left middle frontal gyrus |
|  |  |  |  |  |  |  |
| **Word encoding** | |  |  |  |  |  |
| **word recogn.** | 41144 | -33 -1 6 | .002 | 8.5 | no label | 17.7 % left putamen; 11.5 % left middle temporal gyrus; 11.2 % brain stem; 5.4% left lingual gyrus; 4.8% left pallidum; 4.2% left lateral occipital cortex; 4.2% left temporal occipital fusiform cortex; 4.0% left thalamus; 3.4% left inferior temporal gyrus; 3.2% left temporal fusiform cortex; 2.8% left insular cortex; 2.5% left supramarginal gyrus; 2.3% left angular gyrus; 2.3% left occipital fusiform gyrus; 2.1% left hippocampus; 1.4% left caudate; 1.3% left amygdala; 1.1% left intracalcarine cortex; 0.6% left superior temporal gyrus; 0.4% left planum temporale; 0.2% left precuneus cortex; 0.2% left planum polare |
|  | 25048 | 26 -13 18 | .003 | 6.8 | no label | 22.5% right putamen; 17.4% right Caudate; 8.1% right inferior frontal gyrus pars triangularis; 6.9% right insular cortex; 6.5% right thalamus; 6.4% right middle frontal gyrus; 3.8% right inferior frontal gyrus pars opercularis; 3.4% right frontal opercular cortex; 1.0% right pallidum; 0.9% right precentral gyrus; 0.4% right hippocampus |
|  | 6304 | -55 16 -11 | .04 | 4.2 | left temporal pole | 38.2% left frontal pole; 25.5% left frontal orbital cortex; 20.8% left temporal pole; 13.2% left inferior frontal gyrus pars triangularis; 1.14% left middle frontal gyrus |
|  | 4176 | 22 -81 34 | .06 | 3.8 | right lateral occipital cortex | 38.7% right lateral occipital cortex; 24.3% right cuneal cortex; 23.4% right occipital pole; 7.9% left occipital pole; 5.0% left cuneal cortex |
|  | 8784 | -27 -79 46 | .03 | 5.3 | left lateral occipital cortex | 99.4% left lateral occipital cortex |
|  | 1696 | 36 -63 24 | .03 | 6.0 | no label | 85.4% right lateral occipital cortex; 8.0% right cuneal cortex |
|  | 1288 | -35 46 -1 | .07 | 4.1 | no label | 73.3% left frontal pole; 11.8% left interior frontal gyrus pars triangularis; 6.8% left frontal orbital cortex |
|  | 1160 | 18 6 58 | .05 | 5.1 | no label | 50.3% right superior frontal gyrus; 36.6% right juxtapositional lobule cortex; 10.3% right paracingulate gyrus |
|  | 656 | 28 -57 10 | .09 | 3.8 | no label | 46.3% right precuneus cortex; 40.2% right lingual gyrus |
|  | 600 | 18 -81 -33 | .07 | 4.6 | no label | 49.3% right occipital fusiform gyrus |
|  | 336 | 16 -1 -7 | .08 | 3.8 | right pallidum | 90.5% right pallidum |
|  | 304 | -1 12 40 | .09 | 4.0 | cingulate gyrus anterior division | 97.4% cingulate gyrus anterior division |
|  | 208 | 24 -9 -11 | .09 | 3.5 | right amygdala | 50.0% right amygdala; 42.3 right pallidum |
|  | 160 | -39 18 22 | .10 | 3.0 | left inferior frontal gyrus pars opercularis | 90.0% left inferior frontal gyrus pars opercularis |
|  | 120 | -13 32 48 | .09 | 4.5 | no label | 86.7% left superior frontal gyrus |
|  | 112 | -19 54 16 | .09 | 4.4 | left frontal pole | 100% left frontal pole |
|  | 96 | 20 -43 -15 | .10 | 3.3 | right lingual gyrus | 91.7% right lingual gyrus |

*Note.* Abbreviations: FLE, Frontal lobe epilepsy; FWE, family-wise error rate.

Table S6

*Correlation of fMRI activation of controls during face and word encoding with memory performance.*

| **Correlations in controls: encoding activation with respective recognition accuracies** | | | | | | |
| --- | --- | --- | --- | --- | --- | --- |
|  | Clustersize  [mm³] | Peak | *p*_(FWE)_ | *t* | Peak Region | Cluster Regions [% of cluster in the respective region] |
| **Face encoding** | |  |  |  |  |  |
| **face recogn.** | 1184* | 18 -5 -13 | .02 | 4.1 | right amygdala | 55.4% right hippocampus; 44.6% right amygdala |
| **Word encoding** | | | | | | |
| **word recogn.** | 121240 | -37 8 56 | .008 | 5.0 | left middle frontal gyrus | 11.5% left superior frontal cortex; 10.1% left middle frontal gyrus; 5.7% left putamen; 4.5% left frontal pole; 4.1% left inferior frontal gyrus pars opercularis; 3.7% left caudate; 3.4% left precentral gyrus; 3.0% right putamen; 3.0% left temporal pole; 2.9% left middle temporal gyrus; 2.9% left paracingulate gyrus; 2.7% left insular cortex; 2.7% left thalamus; 2.6% left inferior frontal gyrus pars triangularis; 2.5% right paracingulate gyrus; 2.5% right caudate; 2.4% left supramarginal gyrus; 2.3% left superior temporal gyrus; 1.93% left juxtapositional lobule cortex; 1.8% left angular gyrus; 1.5% left frontal orbital cortex; 2.7% left middle temporal gyrus; 1.4% left superior temporal gyrus; 1.3% right thalamus; 1.3% right pallidum; 1.2% left pallidum; 1.2% right frontal orbital cortex; 1.0% right cingulate gyrus; 0.9% left cingulate gyrus; 0.8% right juxtapositional lobule cortex; 0.7% right superior frontal gyrus; 0.6% left lateral occipital cortex; 0.6% right insular cortex; 0.5% left accumbens; 0.5% left central opercular cortex; 0.4% left planum temporale; 0.3% left frontal opercular cortex; 0.2% left parietal opercular cortex; 0.2% left frontal medial cortex; 0.1% left amygdala; 0.1% left planum polare; 0.1% left subcallosal cortex |
|  | 33280 | 18 -81 -41 | .02 | 5.1 | no label | 22.6% left lingual gyrus; 8.1% left intracalcarine cortex; 5.0% brain-stem; 2.9% right occipital fusiform gyrus; 1.8% left temporal fusiform cortex; 1.6% right lingual gyrus; 1.4% left temporal occipital fusiform cortex; 1.3% right cuneal cortex; 0.9% right supracalcarine cortex; 0.9% right intracalcarine cortex; 0.7% left supracalcarine cortex; 0.4% left occipital fusiform gyrus; 0.4% left parahippocampal gyrus; 0.4% right lateral occipital cortex |
|  | 4904 | -49 -75 26 | .04 | 4.0 | left lateral occipital cortex | 99.2% left lateral occipital cortex |
|  | 312 | -13 -27 -11 | .08 | 4.4 | brain stem | 71.8% brain-stem; 15.4% left parahippocampal gyrus; 7.7% left thalamus; 5.1% left hippocampus |
|  | 256 | 36 2 64 | .09 | 4.0 | right middle frontal gyrus | 59.4% right middle frontal gyrus; 40.6% right precentral gyrus |
|  | 232 | -33 -27 -1 | .09 | 3.9 | no label | 44.8% left putamen; 20.8% left insular cortex; 3.5% left planum polare |
| **verbal learning** | 59304 | -21 -1 -9 | .002 | 7.1 | no label | 10.0% left thalamus; 9.5% left frontal pole; 9.1% left putamen; 8.3% left middle temporal gyrus; 6.6 % left caudate; 5.7% left frontal orbital cortex; 3.9% right caudate; 3.7% left interior temporal gyrus; 3.5% right putamen; 3.1% left hippocampus; 2.8% left insular cortex; 2.3% left temporal fusiform cortex; 2.3% left parahippocampal gyrus; 2.2% left lingual gyrus; 2.1 left precuneus cortex; 2.1% left pallidum; 1.5% left superior temporal gyrus; 1.4% left cingulate gyrus; 1.4% left amygdala; 1.3% right frontal orbital; 1.2% left accumbens; 1.0% right thalamus; 0.8% left lateral occipital cortex; 0.7% left intracalcarine cortex; 0.5% right pallidum; 0.4% left temporal occipital fusiform cortex; 0.4% right insular cortex; 0,4% right accumbens; 0.3% left subcallosal cortex; 0.3% left angular gyrus; 0.2% left postcentral gyrus |
|  | 49256 | -37 32 20 | .01 | 4.6 | left middle frontal gyrus | 22.9% left middle frontal gyrus; 13.6% left frontal pole; 11.1% left interior frontal gyrus pars opercularis; 10.3% left inferior frontal gyrus pars triangularis; 9.4% left superior frontal gyrus; 9.3% left precentral gyrus; 6.3% left cingulate gyrus; 5.5% right frontal pole; 4.4% right cingulate gyrus; 1.9% left paracingulate gyrus; 1.6% left juxtapositional lobule; 1.5% right paracingulate gyrus |
|  | 2240 | -49 -73 28 | .08 | 3.4 | left lateral occipital cortex | 98.2% left lateral occipital cortex |
|  | 400 | -7 14 70 | .09 | 3.1 | left superior frontal gyrus | 96.0% left superior frontal gyrus |
|  | 288 | -45 -41 8 | .09 | 3.4 | no label | 33.3% left planum temporale; 33.3% left supramarginal gyrus; 27.8 left superior temporal gyrus |
|  | 152 | -55 -13 -17 | .09 | 4.3 | left middle temporal gyrus | 100% left middle temporal gyrus |
|  | 48 | 34 32 14 | .09 | 4.2 | no label | 66.7% right inferior frontal gyrus; 16.7% right frontal pole |
|  | 536* | -21 -23 -15 | .03 | 3.5 | left hippocampus | 98.5% left hippocampus |
|  | 80* | 28 -9 -29 | .06 | 3.4 | right hippocampus | 100% right hippocampus |
| **delayed verbal recall** | 25760 | -49 16 36 | .02 | 4.5 | left middle frontal gyrus | 48.6% left middle frontal gyrus; 20.2% left frontal pole; 11.9% left interior frontal gyrus pars opercularis; 8.4% left interior frontal gyrus pars triangularis; 5.4% left superior frontal gyrus; 4.9% left precentral gyrus |
|  | 6936 | -21 2 -7 | .02 | 5.2 | left pallidum | 31.7% left putamen; 22.2% left thalamus; 20.7% left caudate; 11.8% left pallidum; 4.5% left accumbens; 2.9% left frontal orbital cortex; 2.8% left amygdala |
|  | 4848 | -5 36 10 | .04 | 2.7 | left cingulate gyrus | 29.0% right frontal pole; 28.4% right cingulate gyrus; 22.9% left cingulate gyrus; 15.4% right paracingulate gyrus |
|  | 3752 | -13 56 22 | .07 | 3.7 | no label | 67.0% left frontal pole; 18.8% left superior frontal gyrus; 13.7% left paracingulate gyrus |
|  | 1816 | -23 30 -21 | .05 | 4.9 | left frontal orbital cortex | 68.3% left frontal orbital cortex; 26.4% left frontal pole |
|  | 1336 | 12 24 24 | .06 | 4.9 | no label | 70.7% right cingulate gyrus; 24.0% left cingulate gyrus |
|  | 952 | -43 40 -19 | .07 | 3.8 |  | 95.8% left frontal pole |
|  | 624 | -21 -25 -11 | .07 | 4.8 | no label | 55.1 left hippocampus; 12.8% left putamen; 6.4% left parahippocampal gyrus |
|  | 344 | -57 32 -7 | .09 | 3.3 | no label | 62.8% left interior frontal gyrus pars triangularis; 25.6% left frontal pole; 7.0% left frontal orbital cortex |
|  | 88 | -21 20 62 | .10 | 3.2 | left superior frontal gyrus | 100% left superior frontal gyrus |
|  | 256* | -21 -27 -11 | .04 | 4.2 | left hippocampus | 100% left hippocampus |
|  |  |  |  |  |  |  |

*Note.* *Small volume correction in the mesial temporal lobe. Abbreviations: FWE, family-wise error rate.

**Supplementary Information V**

**
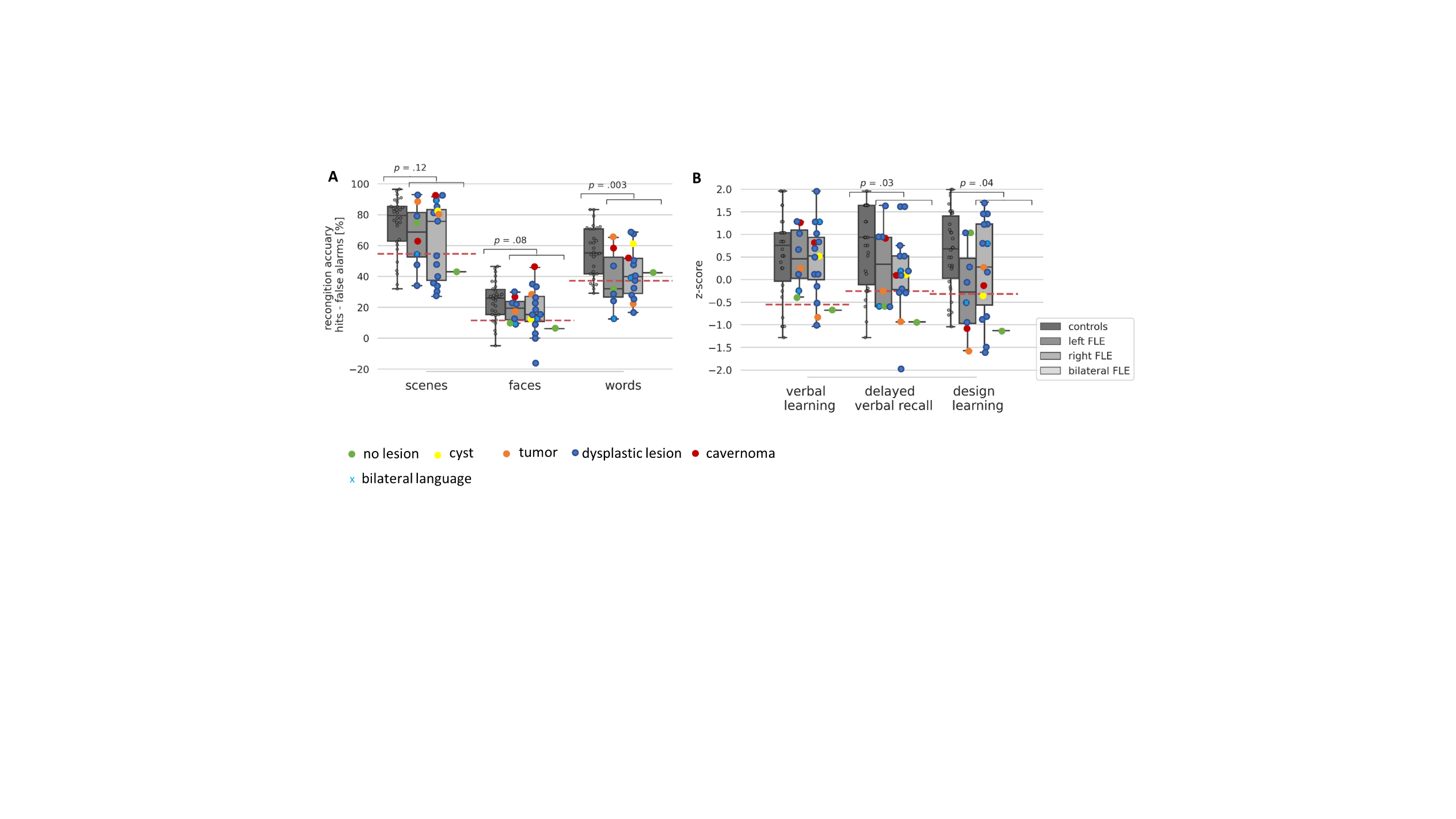
**

*Figure S3.* **Recognition accuracy.** Box plots with overlaid swarm plots, showing **A** the median recognition accuracy (hits – false alarms) for scenes, faces, and words in controls and FLE patients as well as within and between-group variability and **B** verbal learning, delayed verbal recall and design learning z-scores. Coloured points indicate aetiology. The blue “x” mark the two patients with bilateral language laterality. Red dashed line indicates the 16th percentile of the controls’ performance used for splitting patients into subgroups. Abbreviations: FLE, frontal lobe epilepsy.

**
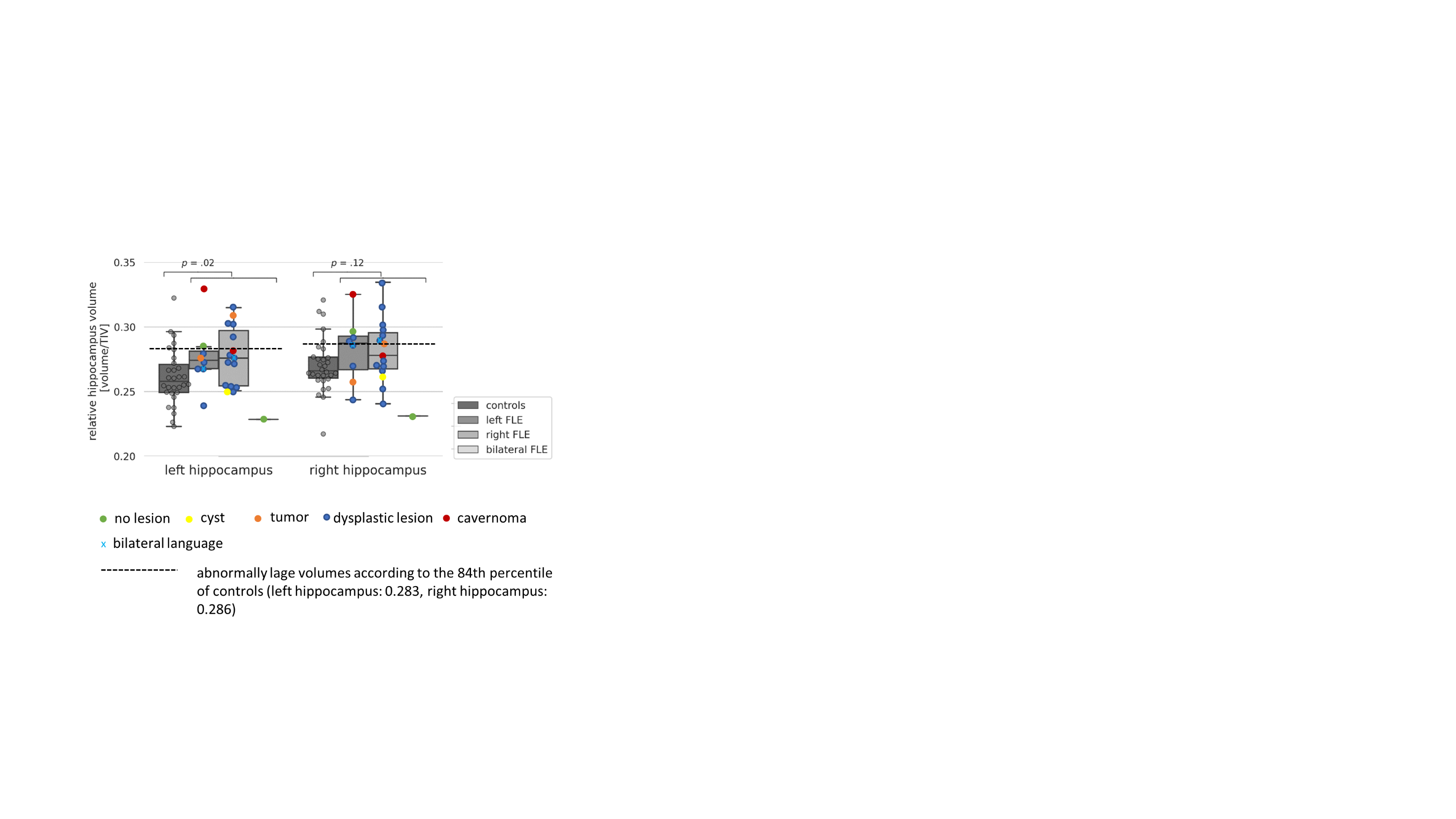
**

*Figure S4.* **Mesial temporal lobe volumes.** Box plot diagrams with overlaid swarm plots, illustrating the relative hippocampus volumes in mm³ in controls and FLE patients. Shown are median values as well as within and between-group variability. Coloured points indicate aetiology. The blue “x” mark the two patients with bilateral language laterality. Abbreviations: FLE, frontal lobe epilepsy; TIV, total intracranial volume.

*
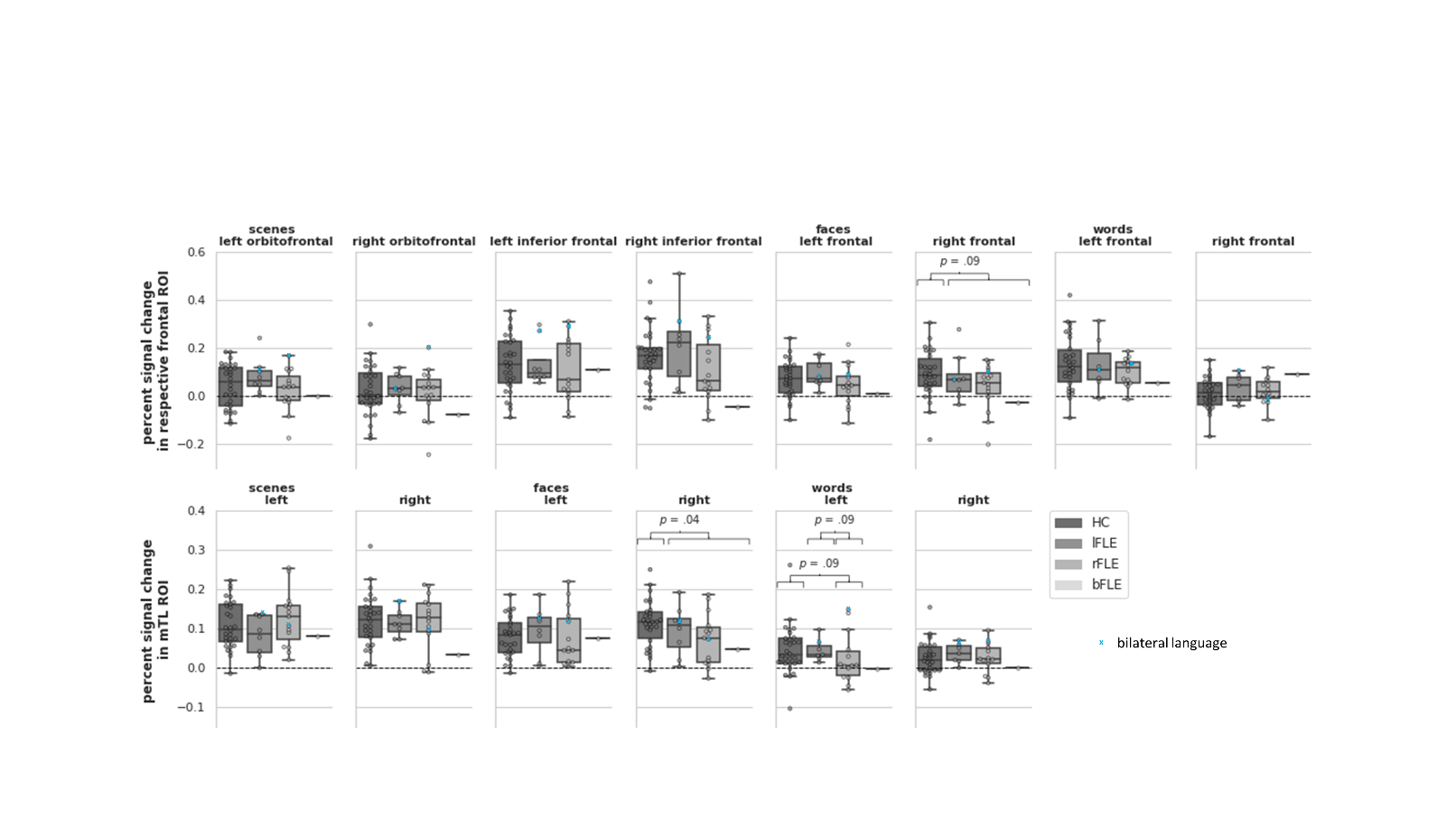
*

*Figure S5.* **fMRI activation encoding versus baseline**. Region of interest analyses of fMRI activation of encoding scenes, faces, and words in FLE patients and controls. Box plot diagrams with overlaid swarm plots. Shown are median values as well as within and between-group variability. *p*-values (uncorrected) correspond to Mann-Whitney U Test. The blue “x” mark the two patients with bilateral language laterality. Abbreviations: bFLE, bilateral frontal lobe epilepsy; FLE, frontal lobe epilepsy; fMRI, functional magnetic resonance imaging; HC, healthy controls; lFLE, left frontal lobe epilepsy; rFLE right frontal lobe epilepsy.

**Supplementary Information VI**

Table S7

*Comparison of demographical and clinical characteristics in normal and low performing FLE patients based on the 16^th^ percentile of controls memory performance.*

|  | Scene recognition | | Face recognition | | Word recognition | | Delayed verbal recall | |
| --- | --- | --- | --- | --- | --- | --- | --- | --- |
| Performance level | normal  n = 13 | low  n = 11 | normal  n = 17 | low  n = 7 | normal  n = 13 | low  n = 9 | normal  n = 18 | low  n = 6 |
| Age in years  [*M (SD)* (range)] | 33.4 (14.1)  (18-65) | 31.5 (11.5)  (18-58) | 31.1 (13.1)  (18-65) | 35.9 (12.0)  (23-58) | 34.3 (16.1)  (18-65) | 30.3 (8.0)  (18-43) | 33.2 (14.1)  (18-65) | 30.3  (7.9)  (23-43) |
| Sex in % [male/female] | 46.2/6  53.8 | 72.7/  27.3 | 47.1/  52.9 | 85.7/  14.3 | 46.2/  53.8 | 66.7/  33.3 | 55.6/  44.4 | 66.7/  33.3 |
| Years of schooling  [*M (SD)* (range)] | 11.1 (1.4)  (10-13) | 10.5 (1.4)  (9-13) | 11.0 (1.5)  (9-13) | 10.4 (1.1)  (9-12) | 10.7  (1.5)  (9-13) | 10.9 (1.4)  (10-13) | 11.1 (1.5)  (9-13) | 10.2  (1.0)  (9-12) |
| Handedness [right/left] | 12/1 | 10/1 | 15/2 | 7/0 | 11/2 | 9/0 | 16/2 | 6/0 |
| Language laterality^a^  [left/bilateral] | 12/1 | 10/1 | 16/1 | 6/1 | 12/1 | 8/1 | 17/1 | 5/1 |
| Laterality of epileptic focus  [left/right/bilateral] | 5/8/0 | 3/7/1 | 6/11/0 | 2/4/1 | 3/9/1 | 4/5/0 | 5/13/0 | 3/2/1 |
| Age at epilepsy onset  [*M (SD)* (range)] | 20.3 (17.2)  (3-62) | 9.0  (8.8)  (0-25)* | 15.5 (17.0)  (0-62) | 14.1 (9.2)  (0.7-25) | 18.4 (18.9)  (0-62) | 11.6 (7.5)  (3-25 | 16.6 (16.3)  (0-62) | 10.8 (9.4)  (0.7-25) |
| Epilepsy duration  [*M (SD)* (range)] | 13.1 (8.5)  (0-29) | 22.5 (7.3)  (12-34)* | 15.6 (9.6)  (0-34) | 21.7 (6.7)  (15-34) | 15.9 (11.7)  (0-34) | 18.8 (4.5)  (12-27) | 16.7 10.4)  (0-34) | 19.6 (3.3)  (15-24) |
| Antiseizure medication load^b^ [*Mdn* (range)] | 2.1  (1.0-5.4) | 2.9  (1.8-5.1)^#^ | 2.5  (1.0-5.4) | 2.9  (2.3-5.1) | 2.6  (1.0-5.4) | 2.5  (1.0-5.1) | 2.6  (1.0-5.4) | 2.5  (1.2-5.0) |
| Aetiology  Focal cortical dysplasia/  unspecified dysplastic  lesion/  cyst/ cavernoma/ tumor/  no lesion | 6/  1/  1/2/2/  1 | 7/  3/  0/0/0/  1 | 11/  1/  1/2/2/  0 | 2/  3/  0/0/0/  2 | 6/  2/  1/2/1/  1 | 6/  1/  0/0/1  /1 | 11/  3/  1/2/1/  0 | 2/  1/  0/0/1/  2 |

*Note.* According to Fisher Exact Test or Mann Whitney U Test there were no group differences (*p*>.1) except for scene recognition and age of epilepsy onset/epilepsy duration; group difference in aetiology were not calculated due to small subgroups. * *p*<.05, # *p*<.1
